# HSPA12A controls cerebral lactate homeostasis to maintain hippocampal neurogenesis and mood stabilization

**DOI:** 10.1038/s41398-023-02573-5

**Published:** 2023-08-14

**Authors:** Jialing Wang, Ting Lu, Yali Gui, Xiaojin Zhang, Xiaofei Cao, Yuehua Li, Chuanfu Li, Li Liu, Zhengnian Ding

**Affiliations:** 1 Department of Anesthesiology, First Affiliated Hospital of Nanjing Medical University, Nanjing, 210029 China; 2 Department of Geriatrics, Jiangsu Provincial Key Laboratory of Geriatrics, the First Affiliated Hospital of Nanjing Medical University, Nanjing, 210029 China; 3 Key Laboratory of Targeted Intervention of Cardiovascular Disease, Collaborative Innovation Center for Cardiovascular Disease Translational Medicine, Nanjing Medical University, Nanjing, 210029 China; 4 Departments of Surgery, East Tennessee State University, Johnson City, TN 37614 USA

**Keywords:** Physiology, Pathogenesis

## Abstract

Mood instability, a subjective emotional state defined as rapid mood oscillations of up and down, is a symptom that occurs in several psychiatric disorders, particularly major depressive disorder and bipolar disorder. Heat shock protein A12A (HSPA12A) shows decreased expression in the brains of schizophrenia patients. However, the causal effects of HSPA12A in any psychiatric disorders are completely unknown. To investigate whether HSPA12A affects mood stability, *Hspa12a-*knockout mice (*Hspa12a*^−^^*/*−^) and wild-type (WT) littermates were subjected to tests of open field, forced swimming, elevated plus maze, and sucrose preference. Cerebral lactate levels were measured in cerebrospinal fluid (CSF). Adult hippocampal neurogenesis (AHN) was assessed by BrdU labeling. We found that acute mood stress increased hippocampal HSPA12A expression and CSF lactate levels in mice. However, *Hspa12a*^−^^*/*−^ mice exhibited behaviors of mood instability (anhedonia, lower locomotor activity, antidepression, and anxiety), which were accompanied by impaired AHN, decreased CSF lactate levels, and downregulated hippocampal glycolytic enzyme expression. By contrast, HSPA12A overexpression increased lactate production and glycolytic enzyme expression of primary hippocampal neurons. Intriguingly, lactate administration alleviated the mood instability and AHN impairment in *Hspa12a*^−^^*/*−^ mice. Further analyses revealed that HSPA12A was necessary for sustaining cerebral lactate homeostasis, which could be mediated by inhibiting GSK3β in hippocampal neurons, to maintain AHN and mood stabilization. Taken together, HSPA12A is defined as a novel regulator of mood stability and exerts therapeutic potential for mood disorder. Our findings establish a framework for determining mood disorder and AHN relevance of cerebral lactate homeostasis.

**Figure:**
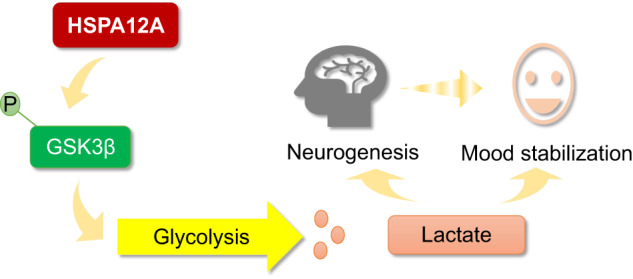
HSPA12A is a novel mood stabilizer through inhibiting GSK3β in hippocampal neurons, thereby sustaining glycolysis-generated lactate to maintain cerebral lactate homeostasis, which ultimately leading to maintenance of hippocampal neurogenesis and mood stabilization.

HSPA12A is a novel mood stabilizer through inhibiting GSK3β in hippocampal neurons, thereby sustaining glycolysis-generated lactate to maintain cerebral lactate homeostasis, which ultimately leading to maintenance of hippocampal neurogenesis and mood stabilization.

## Introduction

Mood instability, a subjective emotional state defined as rapid mood oscillations of up and down, is a symptom that occurs in several psychiatric disorders, particularly major depressive disorder and bipolar disorder (BD) [1]. Also, mood instability is associated with the onset of bipolar disorders and serves as a potential trait marker for BD [2–4]. Mood disorders will not only affect the mental health of patients, but also affect their life quality [5–7]. However, the underlying mechanisms regarding mood stability maintenance is not completely understood.

Appropriate adult hippocampal neurogenesis (AHN) buffers stress responses, whereas impaired AHN has been implicated in anxiety and depression [8, 9]. Many psychoactive drugs, such as antidepressants and mood stabilizers, can boost AHN [10]. Intriguingly, recent studies have demonstrated an active role of lactate in promoting AHN [11–13]. Lactate is an end product of glycolysis and is also implicated in mood regulation. Although different studies have reported conflicting results regarding cerebral lactate levels in individuals with mood disorders (including BD and major depression) [14], lactate administration has been proven to be useful in promoting resilience to stress and treating social avoidance, anxiety, and sleep onset problems associated with depression [12, 15, 16]. These findings suggest that modulating cerebral lactate homeostasis may help stabilize mood and behavior by regulating AHN. However, the regulation of cerebral lactate homeostasis is not yet fully understood.

Heat shock protein A12A (HSPA12A), a novel and atypical member of the heat shock protein 70 (HSP70) family, was cloned from murine aortic atherosclerotic plaque in 2003 [17]. Subsequently, we and others demonstrated high HSPA12A expresses abundantly in the brains of humans and mice under normal conditions [17–19], suggesting a potential involvement of HSPA12A in cerebral function. Indeed, we recently reported that cerebral HSPA12A expression is decreased following ischemic stroke, and that HSPA12A is required for protecting brains against ischemic stroke [20]. Of particular interest, HSPA12A expression is decreased in the brains of patients with schizophrenia [18]. However, it is unknown whether this downregulation of HSPA12A is related to the disease pathogenesis or is a result of the pharmacological treatment of the disorder [18]. Moreover, the causal effects of HSPA12A in any psychiatric disorders are completely unknown. When taken into account that we have recently revealed the regulatory roles of HSPA12A in lactate production in cancer cells [21], we thus hypothesized here that HSPA12A may control lactate homeostasis and thereby modulate neurogenesis and mood stability.

To test this hypothesis, we examined whether HSPA12A affects mood and behavior. We found that mood stress regulated hippocampal HSPA12A expression and cerebrospinal fluid (CSF) lactate content, whereas HSPA12A knockout (*Hspa12a*^*−/−*^) in mice caused mood disorders, impaired AHN, and decreased CSF lactate levels and hippocampal glycolytic enzyme expression. Further analyses revealed that HSPA12A was necessary for sustaining cerebral lactate homeostasis, which could be mediated by inhibiting GSK3β in hippocampal neurons, to maintain hippocampal neurogenesis and mood stabilization. We identified hippocampal HSPA12A as a new mood stabilizer that lends support for its therapeutic potential in mood disorders.

## Materials and methods

### Reagents

FD Rapid Golgi Stain kit was purchased from FD NeuroTechnologies (Columbia, MD). Hoechst 33342 was from Invitrogen (Camarillo, CA). Trizol reagent was from Life Technology (Carlsbad, CA). Normal Goat Serum (NGS), peroxidase conjugated secondary antibody, Cy3- and 488-conjugated secondary antibody were from Jackson lmmunoResearch (West Grove, PA). Bovine serum albumin (BSA) was from Roche (Basel, Switzerland). High glucose Dulbecco’s Modified Eagle’s medium and Neurobasal were from Gibco (Shelton, CT). High-sig ECL western blotting substrate was from Tanon (Shanghai, China). Sodium L-lactate, Lithium chloride (LiCl), and avertin (2,2,2-tribromoethanol) were purchased from Sigma-Aldrich (St Louis, MO). Differentiation-inducing Factor-3 (DIF3) and 5-BrdU were obtained from Santa Cruz Biotechnology (Dallas, TX) and MedChemExpress (Monmouth Junction, NJ). Lactate assay kit was from Jiancheng Biotech (Nanjing, China).

### HSPA12A knockout (*Hspa12a*^−^^*/*−^) mice

*Hspa12a*^−^^*/*−^ mice were generated using Cre-*Lox*P recombinant system as previously described [22]. Briefly, the region of the *Hspa12a* gene containing exons 2–4 was retrieved from a 129/sv BAC clone (BAC/PAC Resources Center, Oakland, CA) using a retrieval vector containing two homologous arms. Exons 2 and 3 were replaced by *lox*P sites flanking a PGK-neo cassette as a positive selection marker. Embryonic stem cells were electroporated with the linearised targeting vector, selected, and then expanded for Southern blot analysis. Chimeric mice (*Hspa12a*^*flox/+*^) were generated by injecting embryonic stem cells into C57BL/6 blastocysts, followed by transferring into pseudo-pregnant mice. To remove the *Hspa12a* gene, the chimeric mice were crossed with EIIa-Cre transgenic mice. The mice were bred at the Model Animal Research Center of Nanjing University and were maintained in the Animal Laboratory Resource Facility of the same institution. All experiments conformed to the Guide for the Care and Use of Laboratory Animals published by the US National Institutes of Health (NIH Publication, 8th Edition, 2011) and international guidelines on the ethical use of animals. The animal care and experimental protocols were under the regulation and approval of Committee on Animal Care of Nanjing University and Nanjing Medical University. Male mice aged at 8–10-week with C57BL/6 background were used in the experiments. Mice were randomly allocated to each experimental group. For tissue collection, mice were sacrificed by overdose anaesthesia with pentobarbital sodium (150 mg/kg, intraperitoneal injection) and cervical dislocation.

### Acute restraint swimming stress (ASS)

ASS for 15 min is an effective physical and psychological stress that causes emotional alterations in rodents [23, 24]. C57BL/6 mice were individually placed in a water tank (H×D: 30 × 10 cm) filled with 24–25 °C water to a depth of 25 cm. After swimming stress for 15 min, mice were sacrificed for collecting frontal cortex and hippocampus. The water was renewed for each mouse with clean water.

### Chronic restraint stress (CRS)

CRS was induced in mice as previous methods [25, 26]. Briefly, C57BL/6 mice were restrained in a 50 ml centrifuge tube for 2 h per day for 14 days. A hole was created in the front end of the tube to allow the mice to breathe, and another hole was created in the lid to allow the tail of the mouse to pass through. The time of the restraint stress always carried out during 10:00–12:00 am.

### Behavioral tests

Behavioral tests were performed on *Hspa12a*^−^^*/*−^ mice and WT littermates (the floxed-*Hspa12a* without Cre-transgenine) of 8–10-week of age, and the sample size was more than 5 samples per group according to the methods described previously [9, 27–33]. The interval between different behavioral tests was at least 3 d. The nonstressful tests were performed first: sucrose preference, open field test, elevated plus maze, and self-grooming. The stressful tests were performed last: forced swim test and tail suspension tests. All tests were measured by two trained observers who were blinded to the genotypes and treatment of mice. The test protocols were as followings.

### Sucrose preference test

This test was performed according to previous methods. Mice were individually housed with free access to food and two bottles of liquid, one containing water and the other containing 1% sucrose solution. After 72 h of habituation, both liquid buttles were removed at 18 pm to restrict water overnight. At next 6 am, the bottles were preweighted and put back in reversed locations. The bottles were weighed again 24 h later to determine liquid consumption. To avoid any confounding effect of side preference, the position of the sucrose and water bottles was exchanged every 6 h. Sucrose preferences were calculated as follows: sucrose consumption/(sucrose consumption+water consumption).

### Open field test

The apparatus was a square arena (W×D×H: 50 × 50 × 50 cm). A mouse was placed in the center and allowed to freely explore the arena for 20 min. The arena was divided equally to 16 squares, including areas of center (the inner 4 squares), corner (4 squares), and round (the left 8 squares). The digital camera was 160 cm above the bottom of apparatus. The movements were recorded using a digital camera during test. The total distance moved, averaged moving speed, time spent and entries in the arenas were analyzed using an automatic monitoring system (TopScan, CleverSys, Inc. Reston, VA).

### Elevated plus maze

This test was performed according to previous methods. The elevated plus maze consisted of two open arms (30 × 5 cm, wall-free) and two enclosed arms (30 × 5 cm) surrounded by 15 cm high walls. The maze was elevated 50 cm from the ground. Mouse was placed in the center of the apparatus facing one of the closed arms and allowed to freely explore the maze for 5 min. An arm entry was scored when four legs were within the arm. Time spent in open arms were recorded using an automatic monitoring system (TopScan, CleverSys, Inc. Reston, VA).

### Forced swim test

Mouse was placed in a water tank (H × D: 30 × 10 cm) filled with water (24 ± 1 °C) to a depth of 25 cm. Mice were forced to swim 6 min, and the immobile time and events were recorded only during the last 4 min. Immobility was defined as floating without any movements except for a single limb paddling to keep balance in the water.

### Tail suspension test

This test was performed according to previous methods. Mouse was suspended for 6 min by taping the tip of tail (1 cm) to a bar and the nose tip was at least 15 cm above the floor. The immobility time and events were measured only during the last 4 min. The immobility was defined as hanging by the tail without engaging in any active upward movements of the entire body.

### Self-grooming test

The spontaneous grooming behaviours were recorded when mice were placed individually in a clean, empty cage without bedding, using methods previously described. Each mouse was given a 10 min habituation period and then rated for 10 min for cumulative events of grooming all body regions. The test session was videotaped and scored later

### Lactate and lithium treatment in mice

For lactate treatment, mice were injected intraperitoneally with lactate (1 mg/g body weight) for 21 consecutive days according to previous studies [34]. For LiCl treatment, mice drank water containing LiCl (600 mg/L) based on the health concern and drug effectiveness described previously [35]. After treatment, the mice received behavioral tests. After tests, hippocampi were collected and processed for cryosection for the immunofluorescence staining.

### BrdU incorporation

Mice were injected intraperitoneally with BrdU (100 μg/g body weight) for three times every two hours according to our previous methods [20]. Two hours after the last injection, brains at hippocampal levels were collected and processed for cryosection for the immunofluorescence staining.

### Primary hippocampal neuron isolation, culture, and treatment

Primary hippocampal neurons were isolated from the hippocampi of newborn rats (0 d) according to previous methods[20] . Briefly, after cut into pieces, the hippocampi were digested in 37 °C incubator for 15 min followed by slowly blowing with a straw for 6–7 times. Subsequently, the isolated cells were centrifuged, resuspended, and transferred to the petri-dish. After 4 h of culture with high glucose DMEM, the medium was changed to Neurobasal medium containing 2% B27 supplement.

To overexpress HSPA12A, primary hippocampal neurons were infected with HSPA12A-adenovirus (Ad-*Hspa12a*). The neurons infected with empty adenovirus served as negative controls (Ad-NC). To activate GSK3β, the neurons were treated with DIF3 (30 μM) for 3 h according to previous studies [36, 37]. For lactate treatment, the primary neurons were treated with lactate (5 mM) for 24 h according to previous studies [38].

### Lactate measurement

Cerebral lactate levels were measured in CSF. The CSF were colleced as the methods decirbed previously [39]. The surgery of CSF collection was shown in Supplemental video. Briefly, mice were anesthetized with intraperitoneal injection with avertin (200 mg·kg^−1^) and the withdrawal response to toe pinch was performed to assure adequate anesthesia. After cut skin across the back of the neck down to the middle of the skull between the ears and eyes of mouse, the dura over the cisterna magna is exposed after pulling apart the tissues. Subsequently, glass capillary was punctured to the cisterna magna to collect CSF. The CSF lactate contents were measured using the assay kit.

To measure lactate generation of primary hippocampal neurons, the extracellular lactate contents in medium were measured using the assay kit.

### Immunoblotting and immunofluorescence staining

Immunoblotting was performed according to our previous methods [40, 41]. Birefly, equal amounts of extracted proteins were separated on SDS-PAGE and transferred onto Immobilon-P membranes (Millipore). The membranes were probed with appropriate primary antibodies followed by incubation with peroxidase-conjugated secondary antibodies. The signals were detected by Chemiluminescent Substrate. To control for lane loading, the same membranes were probed with anti-GAPDH. The signals were quantified by scanning densitometry and the results from each experimental group were expressed as relative integrated intensity compared with that of controls.

Immunofluorescence staining was performed according to our previous methods [40, 41]. Briefly, brains at hippocampus levels were collected and processed for cryosection at 4 μm. After penetrated cell membrane with 0.3% triton and blocked with 5% normal goat serum, the cryosections were incubated with the indicated primary antibodies overnight at 4 °C followed by incubation with Cy3- or FITC-conjugated appropriate secondary antibodies. DAPI was used to counterstain the nuclei. The staining was observed using a fluorescence microscope at a magnification of 400× (Olympus, Tokyo, Japan).

For immunoblotting and immunofluorescence staining analyzed, sample size was more than 3 samples per group, and the antibodies used in the experiments were listed in Supplemental table 1.

### Golgi staining

Golgi staining was performed using the FD Rapid Golgi Stain kit according to the manufacturer’s protocol. In brief, freshly dissected brains were immersed in the mixture of solutions A and B for 2 weeks and transferred to solution C for 5 days at room temperature. The brains were sliced at a thickness of 100 μm. The sections were mounted to gelatin-coated microscope slides, placed in the mixture of Solutions D and E for 10 min. The staining was observed using a microscope (Olympus, Japan) at 100× magnification, and images of granule neurons in the dentate gyrus (80 neurons per genotype) were obtained. The total dendrite length and spine density was quantified using the Cellsens Dimention 1.15 software, (Olympus, Japan).

### Re-expression of HSPA12A in hippocampus of *Hspa12a*^−^^*/*−^ mice

We re-expressed HSPA12A in hippocampus of *Hspa12a*^−^^*/*−^ mice by delivering HSPA12A-adenovirus to hippocampus using stereotaxic apparatus according to previous methods [42]. Briefly, mice were anaesthetized with inhalation of 1.5–2% isoflurane and the withdrawal response to toe pinch was used to indicate the adequate anesthesia. After setting the coordinates of the hippocampus on the instrument, a volume of 1 μl of HSPA12A-adenovirus solution (3 × 10^10^ PFU) was injected to hippocampus at a rate of 200 nl/min via microinjector with RWD stereotaxic apparatus (Life science, USA). The infusion needle was left in place for an additional 5 min to allow for diffusion. Bilateral hippocampus was injected with HSPA12A-adenovirus solution. The *Hspa12a*^−^^*/*−^ mice injected with empty control adenovirus served as controls.

### Statistical analysis

**Fig. 1:**
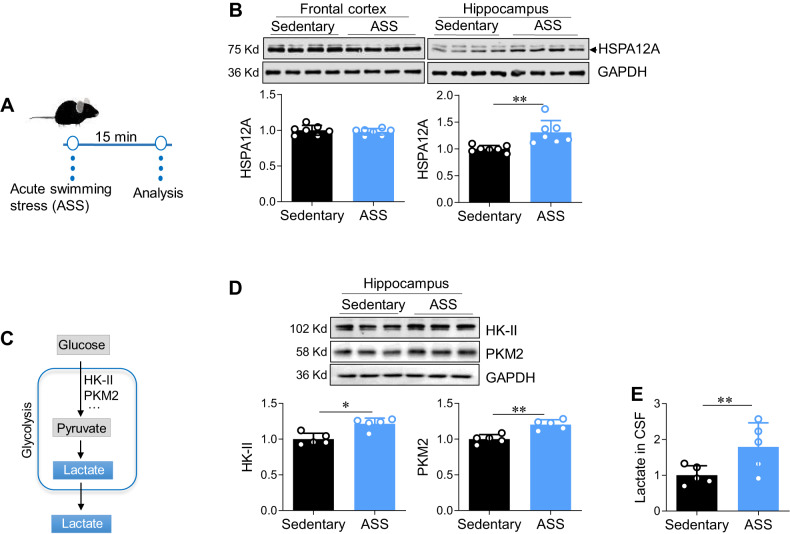
HSPA12A was upregulated in hippocampus following acute psychological stress. **A** Experimental protocol. **B** HSPA12A expression. After ASS, the indicated tissues were collected for immunoblotting against HSPA12A. The blots against GAPDH served as loading controls. *n* = 7/group. **C** Brief illustration of glycolysis pathway. **D** Glycolysis-related gene expression. After ASS, the indicated gene expression was analyzed in hippocampus by immunoblotting analysis. *n* = 5/group. **E** Lactate in cerebrospinal fluid (CSF). After ASS, CSF was extracted from the mice for lactate content measurement. *n* = 5/group. Data are mean ± SD, **P < 0.01 and *P < 0.05 by Student’s two-tailed unpaired *t* test or Mann–Whitney U test.

**Fig. 2:**
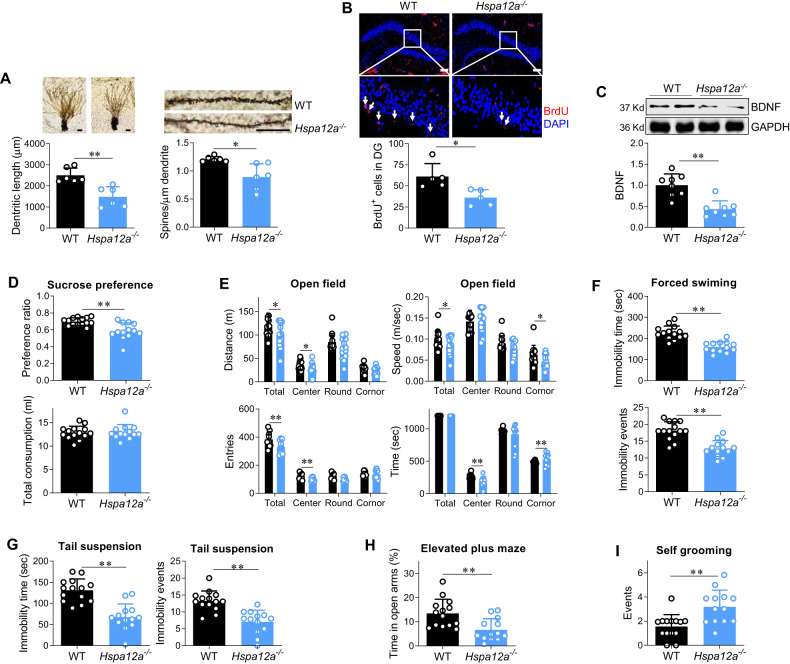
Hspa12a^−^/^−^ mice displayed impairment of neurogenesis, neuronal spinogenesis in hippocampus and displayed mood disorder. **A** Neuronal dendrite length and dendrite spine density. Neuronal dendrite length (Scale bar = 50 μm) in dentate gurus and spine density (Scale bar = 20 μm) of neuronal dendrite in dentate gurus was analyzed using Golgi staining. *n* = 6/group. **B** Adult hippocampal neurogenesis (AHN). AHN was examined in granular layer and interspace of dentate gyrus (DG) by BrdU incorporation. DAPI was used to counter stain nuclei. The images showed the representative staining in DG, and the boxed areas were magnified in the down panels. *n* = 5/group. Scale bar = 100 μm. **C** BDNF expression. The expression of BNDF was examined in hippocampus by immunoblotting. *n* = 8/group. **D** Sucrose preference test. The total liquid consumption and sucrose preference ratio were measured in 24 h duration in mice. **E** Open field test. The time spent, distance travelled, moving speed and entries in each area of an open field were recorded within 20 min duration. **F** Forced swimming test. Immobility episodes and time were recorded within 4 min duration of test. **G.** Tail suspension test. Immobility episodes and time were recorded within 4 min duration of test. **H** Elevated plus maze test. The time spent in open arm was recorded within 5 min of test. **I** Self-grooming test. The events of self-grooming were recorded within 10 min of test. Data are mean ± SD, ^**^P < 0.01 and ^*^P < 0.05 by Student’s two-tailed unpaired *t* test or Mann–Whitney U test test, *n* = 14 for WT group and *n* = 13 for *Hspa12a*^*−/−*^ group.

**Fig. 3:**
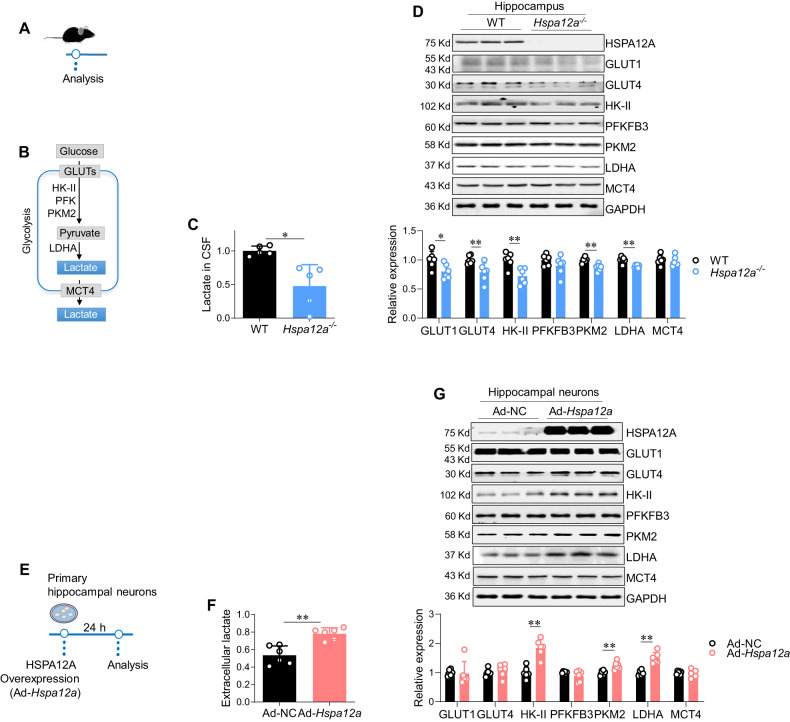
HSPA12A was required for maintaining cerebral lactate homeostasis and promoting lactate generation by hippocampal neurons. **A** Experimental protocol of mice. **B** Brief illustration of glycolysis pathway. **C** Lactate in cerebrospinal fluid (CSF). CSF was extracted from the mice and lactate content was measured. *n* = 5/group. **D** Hippocampal expression of glycolysis-related genes. Hippocampi were collected for measuring the indicated gene expression by immunoblotting analysis. *n* = 6/group. **E** Experimental protocol of primary hippocampal neurons. **F** Extracellular lactate of neuron cultures. After HSPA12A overexpression for 24 h, culture medium of primary hippocampal neurons was collected for measuring lactate contents. *n* = 6/group. **G** Expression of glycolysis-related genes in hippocampal neurons. After HSPA12A overexpression for 24 h, primary hippocampal neurons were collected for measuring the indicated gene expression by immunoblotting analysis. *n* = 6/group. Data are mean ± SD, ^**^P < 0.01 and ^*^P < 0.05 by Student’s two-tailed unpaired *t* test or Mann–Whitney U test.

**Fig. 4:**
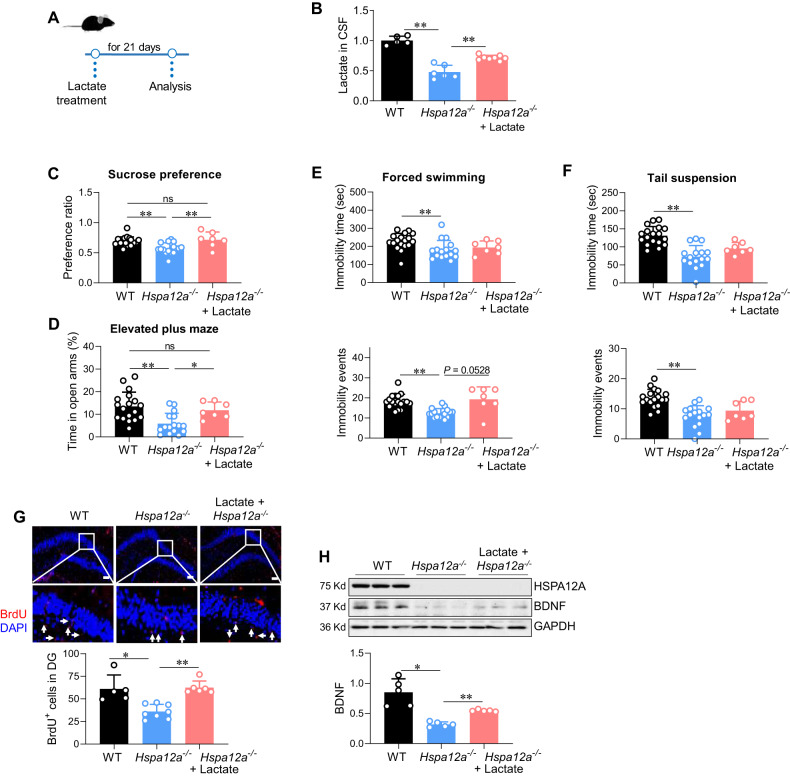
Lactate administration alleviated mood disorder and rescued the impairment of hippocampal neurogenesis in Hspa12a^−^/^−^ mice. **A** Experimental protocol. After lactate treatment for 21 days, the following tests were performed. **B** Lactate levels in cerebrospinal fluid (CSF). *n* = 5 for WT group, *n* = 6 for *Hspa12a*^−^^*/*−^ group and *n* = 8 for lactate-treated group. **C–F** Behavioral tests. *n* = 18 for WT group, *n* = 16 for *Hspa12a*^−^^*/*−^ group, and *n* = 7 for lactate-treated *Hspa12a*^−^^*/*−^ group. **G** Adult hippocampal neurogenesis (AHN). AHN was examined in granular layer and interspace of dentate gyrus (DG) by BrdU incorporation. DAPI was used to counter stain nuclei. The images showed the representative staining in dentate gyrus, and the boxed areas were magnified in the down panels. *n* = 5, 8 and 6 for WT, *Hspa12a*^−^^*/*−^, and lactate-treated *Hspa12a*^−^^*/*−^ group, respectively. Scale bar=100 μm. **H** BDNF expression. The expression of BNDF was examined in hippocampus by immunoblotting. *n* = 5/group. Data are mean ± SD; ^**^P < 0.01 and ^*^P < 0.05 by one-way ANOVA followed by post-hoc test (**C, F, G**) and Kruskal–Wallis test (**B,** **D–E, H**). ns no significance.

**Fig. 5:**
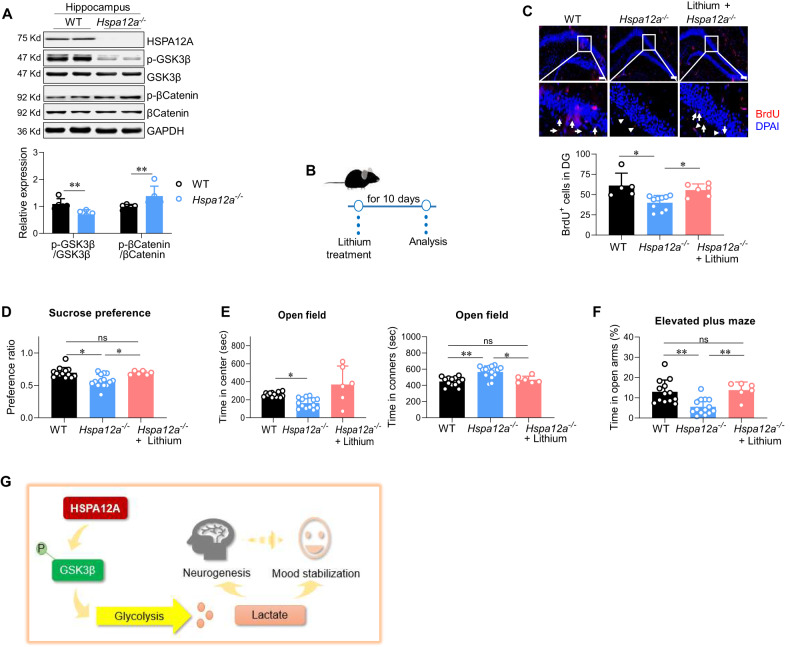
HSPA12A regulated GSK3β pathway, and GSK-3β inhibitor lithium rescued mood disorder and hippocampal neurogenic impairment in Hspa12a^−^/^−^ mice. **A** Immunoblotting in hippocampus. The indicated gene expression was examined in hippocampus by immunoblotting analysis. *n* = 5/group. **B** Experimental protocol. Following lithium treatment for 10 days, the following experiment were performed. **C** Adult hippocampal neurogenesis (AHN). AHN was examined in granular layer and interspace of dentate gyrus (DG) by BrdU incorporation. DAPI was used to counter stain nuclei. The images showed the representative staining in dentate gyrus, and the boxed areas were magnified in the down panels. *n* = 5, 10 and 5 for WT, *Hspa12a*^−*/*−^, and lithium-treated *Hspa12a*^−^^*/*−^ group, respectively. Scale bar= 100 μm. **D–F** Behavioral tests. *n* = 13 for WT group, *n* = 14 for *Hspa12a*^−^^*/*−^ group, and *n* = 6 for lithium-treated group. ns, no significance. **G** Mechanistic scheme. HSPA12A was required for sustaining cerebral lactate homeostasis to maintain hippocampal neurogenesis and mood stabilization through inhibiting GSK3β in hippocampal neurons. Hippocampal HSPA12A is identified as new regulator of mood behaviors that lends support for its therapeutic potential in mood stabilization. Data are mean ± SD, ^**^P < 0.01 and ^*^P < 0.05 by Mann–Whitney U test (**A**) and Kruskal–Wallis test (**D–F**).

Results were expressed as the mean±standard deviation and were analysed parametrically. All data sets were first tested for normality and homogeneity before choosing the statistical test. Data were consistent with normality and homogeneity of variance, groups were compared using Student’s two-tailed unpaired *t-*test (Figs. 1B, D, 2B, D–G, Fig. S2, Fig. 3C, F, G, Figs. S3, S4C, S7, S8B), or two-way analysis of variance analysis followed by Tukey test as a post-hoc test (Fig. 4C, F, G, Figs. S6B, C, S9A–F, S10B, C, S11B, C). Some data were not consistent with normality and homogeneity of variance, Mann–Whitney U test (Figs. 1B, D, 2A, C–F, H–I, 3D, F, 4B, 5B) and Kruskal–Wallis (Figs. 4D, E, H, 5D–G, S12A–C) were used for analyses. *P* < 0.05 was considered statistically significant.

## Results

### Mood stress affects hippocampal HSPA12A expression and CSF lactate levels in mice

The hippocampus and frontal cortex play critical roles in modulating mood stability [43, 44]. To determine whether HSPA12A is expressed in neurons of these areas, immunofluorescence staining was performed. In both the hippocampus and frontal cortex of mice, HSPA12A was present in cells that were positive for β-tubulin III, a neuronal marker (Fig. S1).

To test whether HSPA12A is involved in mood regulation, we examined HSPA12A expression after ASS for 15 min (Fig. 1A), which represents an effective physical and psychological stress that causes emotional alterations in rodents [23, 24]. HSPA12A expression was upregulated in the hippocampus but remained unchanged in the frontal cortex in mice that underwent ASS, compared to sedentary controls (Fig. 1B). After ASS, the hippocampal expression of the glycolytic enzyme hexokinase 2 (HK-II) and pyruvate kinase M2 (PKM2) was also upregulated (Fig. 1C, D), and the CSF levels of lactate (an end product of glycolysis) were increased (Fig. 1E). ASS did not change sucrose consumption of sucrose preference test and time spent in open arms of elevated plus maze (Fig. S2A). By contrast to ASS, chronic stress reduced sucrose consumption in sucrose preference test, decreased HSPA12A and PKM2 expression in hippocampus, lowered lactate levels in CSF (Fig. S2B–E). Moreover, we found that ASS upregulated HSP27 and HSP70 expression whereas chronic stress did not change HSP27 and HSP70 expression in hippocampus of mice (Fig. S3). These findings suggest a potential involvement of hippocampal HSPA12A and cerebral lactate homeostasis in mood regulation.

### HSPA12A knockout impairs AHN and causes depression and anxiety in mice

#### *Hspa12a*^−*/*−^ mice exhibit AHN impairment

To determine whether HSPA12A modulates mood stability, *Hspa12a*^−^^*/*−^ mice and WT littermates were used. The deletion of hippocampal HSPA12A expression in *Hspa12a*^−^^*/*^^−^ mice is shown in Fig. S4A, B. Adult (8-week-old) *Hspa12a*^−^^*/*^^−^ mice exhibited similar brain morphology and brain weight as WT mice (Fig. S4C). However, Golgi staining revealed decreases of dendritic length and dendritic spines of *Hspa12a*^−^^*/*^^−^ hippocampal neurons compared to WT controls (Fig. 2A). Remarkably, *Hspa12a*^−^^*/*^^−^ mice exhibited less BrdU labeling in hippocampal dentate gyrus (DG) areas than WT controls (Fig. 2B). Also, *Hspa12a*^−^^*/*^^−^ mice exhibited lower expression level of hippocampal brain-derived neurotrophic factor (BDNF), an important factor for neurogenesis [45], than WT mice (Fig. 2C). Immunostaining showed a less BDNF expression in neurons in hippocampus of *Hspa12a*^−^^*/*^^−^ mice than WT controls, and the expression of BDNF was present in both neuronal body and dendrites of hippocampal neurons (Fig. S5A, B). These results indicate that HSPA12A knockout impairs neurogenesis and neuronal spinogenesis in the hippocampus of mice.

#### *Hspa12a*^−^^*/*^^−^ mice displayed behaviors of mood instability

To determine whether HSPA12A affects behaviors, behavioral tests were performed. Diminished hedonic response is one of the core symptoms of major depression [9], and the sucrose preference test showed that *Hspa12a*^−^^*/*^^−^ mice consumed less sucrose solution than WT controls (Fig. 2D). Similarly, *Hspa12a*^−^^*/*^^−^ mice exhibited lower locomotor activity, observed as a smaller distance and lower speed in the open field test (Fig. 2E). These findings indicate that HSPA12A knockout causes a depression-like mood. However, two other depression-related behavioral tests (the forced swim test and tail suspension test) [27, 28] revealed shorter immobility time and fewer immobility events in *Hspa12a*^−^^*/*^^−^ mice compared to those in WT controls (Fig. 2F, G), suggesting an antidepressive effect of HSPA12A knockout.

Next, we analyzed anxious-like behavior in mice using tests of open field, elevated plus maze, and repeated self-grooming, according to previous studies [29–33]. *Hspa12a*^−^^*/*^^−^ mice spent less time in the central area and more time in the corners of the open field than WT controls (Fig. 2E). Less entries into the central area were also observed in *Hspa12a*^−^^*/*^^−^ mice (Fig. 2E). Similarly, *Hspa12a*^−^^*/*^^−^ mice spent less time in the open arms of the elevated plus maze than WT controls (Fig. 2H). Additionally, more self-grooming events were observed in *Hspa12a*^−^^*/*^^−^ mice than WT controls (Fig. 2I). These findings demonstrate that *Hspa12a*^−^^*/*^^−^ mice exhibit anxiety-like behaviors.

Altogether, HSPA12A knockout resulted in multiple aspects of mood instability in mice, including anhedonia, lower locomotor activity, antidepression, and anxiety-like behaviors.

### HSPA12A is required for maintaining lactate homeostasis in CSF and lactate production in hippocampal neurons

Because hippocampal HSPA12A upregulation was accompanied by increased CSF lactate levels after ASS (Fig. 1B, E), we investigated whether cerebral lactate is involved in the regulatory effect of HSPA12A on behavior (Fig. 3A, B). CSF lactate levels were greatly decreased in *Hspa12a*^−^^*/*^^−^ mice compared to WT mice (Fig. 3C). To determine whether this decrease in CSF lactate is related to the hippocampus, we analyzed the expression of glycolysis-related genes for lactate production. The hippocampus of *Hspa12a*^−^^*/*^^−^ mice expressed significantly lower levels of glucose transporter 1 and 4 (GLUT1 and GLUT4), HK-II, PKM2, and lactate dehydrogenase A (LDHA) than those of WT controls (Fig. 3D).

To further determine the role of HSPA12A in lactate production of hippocampal neurons, primary rat hippocampal neurons were isolated and overexpressed with HSPA12A (Fig. 3E). HSPA12A overexpression (Ad-*Hspa12a*) increased extracellular lactate levels of hippocampal neurons compared to negative controls (Ad-NC) (Fig. 3F), suggesting that HSPA12A increases lactate production by glycolysis in hippocampal neurons. Indeed, Ad-*Hspa12a* hippocampal neurons exhibited higher expression of the glycolytic enzymes HK-II, PKM2, and LDHA than Ad-NC (Fig. 3G).

### Lactate treatment mitigates mood instability and AHN impairment in *Hspa12a*^*-/-*^ mice

It is interesting to found that hippocampal HSPA12A upregulation was accompanied by increases in CSF lactate levels after ASS (Fig. 1B, E), whereas HSPA12A ablation caused mood instability and decreased CSF lactate levels (Figs. 2D–I and 3C). These findings motivated us to investigate whether decreased CSF lactate levels mediate the development of mood instability in *Hspa12a*^−^^*/*^^−^ mice. To this aim, lactate was peripherally administered by intraperitoneal injection for 21 days, in accordance with previous studies [34] (Fig. 4A). After lactate administration, *Hspa12a*^−^^*/*^^−^ mice exhibited increased CSF lactate levels (Fig. 4B). Of note, lactate administration rescued the decreased sucrose preference in *Hspa12a*^−^^*/*^^−^ mice (Fig. 4C). Additionally, the decreased time spent in the open arms of the elevated plus maze was rescued by lactate treatment in *Hspa12a*^−^^*/*^^−^ mice (Fig. 4D). No differences in immobility in the forced swim test and the tail suspension test were detected between lactate-treated and untreated *Hspa12a*^−^^*/*^^−^ mice (Fig. 4E, F).

Next, the effects of CSF lactate homeostasis on impaired AHN in *Hspa12a*^−^^*/*^^−^ mice were examined. After lactate administration, *Hspa12a*^−^^*/*^^−^ mice exhibited increased CSF lactate levels and more BrdU labeling and more neuronal dendrite spinogenesis in hippocampal dentate gyrus areas than untreated *Hspa12a*^−^^*/*^^−^ mice (Fig. 4G, S6A–C). Moreover, after lactate treatment, the downregulated BDNF expression in the hippocampi of *Hspa12a*^−^^*/*^^−^ mice was also improved (Fig. 4H). In the cultured primary hippocampal neurons, we found that lactate administration upregulated HSPA12A expression (Fig. S7).

Together, these findings indicate that the maintenance of cerebral lactate homeostasis mediates the regulatory effects of HSPA12A on behavior and AHN.

### HSPA12A maintains lactate production in hippocampal neurons by inhibiting GSK3β

Lactate production by glycolysis is regulated by a broad set of kinases. Considering that we have recently reported that HSPA12A negatively regulates GSK3β activity [20], we investigated whether GSK3β plays a role in HSPA12A-regulated lactate production in hippocampal neurons (Fig. S 8A). Consistent with our previous findings in global brain tissues [20], the hippocampi of *Hspa12a*^−^^*/*^^−^ mice exhibited lower GSK3β phosphorylation levels (Ser9) than WT controls (Fig. 5A, B). By contrast, hippocampal neurons with HSPA12A overexpression exhibited higher GSK3β phosphorylation levels than Ad-NC (Fig. S8B). Beta-catenin, a downstream target of GSK3β, showed higher phosphorylation levels in *Hspa12a*^−^^*/*^^−^ hippocampus (Fig. 5A) but remained unchanged in Ad-*Hspa12a* hippocampal neurons (Fig. S8B).

We next examined whether GSK3β mediates HSPA12A-induced lactate production in hippocampal neurons. Primary hippocampal neurons were treated with DIF3, a GSK3β activator [36, 37] (Fig. S9A). In Ad-*Hspa12a* hippocampal neurons, when DIF3 abolished the increase of GSK3β phosphorylation levels, the increase of extracellular lactate levels and upregulated expression of HK-II, PKM2, and LDHA were also ablated (Fig. S9B, C). Also, after treatment with GSK3β inhibitor lithium, *Hspa12a*^−^^*/*^^−^ mice displayed higher levels of CSF lactate content and glycolytic enzyme PKM2 and LDHA expression than untreated *Hspa12a*^−^^*/*^^−^ mice (Fig. S9D–F). Together, these findings indicate that HSPA12A stimulates lactate production in hippocampal neurons by inhibiting GSK3β.

### The GSK3β inhibitor lithium improves mood instability and AHN impairment in *Hspa12a*^−*/−*^ mice

The effects of GSK3β inhibition on HSPA12A-maintained lactate production in hippocampal neurons prompted us to determine the effects of GSK3β inhibition on depression and anxiety behaviors in *Hspa12a*^−^^*/*^^−^ mice. To this aim, mice were treated with the GSK3β inhibitor lithium or vehicle (normal saline) for 10 days (Fig. 5B). After lithium treatment, *Hspa12a*^−^^*/*^^−^ mice displayed more BrdU-labeled cells and neuronal spinogenesis in hippocampal dentate gyrus areas than untreated *Hspa12a*^−^^*/*^^−^ mice (Fig. 5C, S10A–C). Notably, lithium rescued anhedonia in *Hspa12a*^−^^*/*^^−^ mice, which exhibited an increased sucrose preference when compared to untreated *Hspa12a*^−^^*/*^^−^ mice (Fig. 5D). Additionally, lithium rescued the decreased time in the open arms of the elevated plus maze, the decreased time in the central area and the increased time in the corners of the open field in *Hspa12a*^−^^*/*^^−^ mice, when compared to untreated *Hspa12a*^−^^*/*^^−^ mice (Fig. 5E, F).

### Re-expression of HSPA12A in hippocampus of *Hsp12a*^−^^*/*^^−^ mice reversed the decrease of CSF lactate levels, alleviated AHN impairment, and improved mood instability

Finally, we investigated whether re-expression of HSPA12A in hippocampus could rescue the abnormalities in *Hspa12a*^−^^*/*^^−^ mice. To this aim, HSPA12A was re-expressed in hippocampus of *Hspa12a*^−^^*/*^^−^ mice by delivering HSPA12A-adenovirus to hippocampus using stereotaxic apparatus according to previous methods [42]. Immunoblotting confirmed the re-expression of HSPA12A in hippocampus of *Hspa12a*^−^^*/*^^−^ mice (Fig. S11A). Meanwhile, re-expression of HSPA12A rescued the decreases of HK II, PKM2, and LDHA expression in hippocampus and lactate content in CSF of *Hspa12a*^−^^*/*^^−^ mice (Fig. S11A, B). Also, HSPA12A re-expression improved GSK3β phosphorylation levels in hippocampus of *Hspa12a*^−^^*/*^^−^ mice (Fig. S11A). Moreover, re-expression of HSPA12A increased BrdU labeling in hippocampal dentate gyrus areas in *Hspa12a*^−^^*/*^^−^ mice (Fig. S11C). Importantly, following HSPA12A re-expression, *Hspa12a*^*−/−*^ mice displayed improvements of sucrose preference, immobility time in forced swimming and tail suspension tests, respectively, when compared to control *Hspa12a*^−^^*/*^^−^ mice (Fig. S12A–C).

## Discussion

The present study has identified HSPA12A as a stabilizer of mood and behavior to alleviate mood disorders by maintaining GSK3β-mediated glycolysis in hippocampal neurons and thus cerebral lactate homeostasis (Fig. 5G). Therefore, hippocampal HSPA12A represents a potential therapeutic target for mood instability.

Mood disorders were a kind of psychiatric illness that can simultaneously affect one’s health and life, including sleep changes, cognitive symptoms, fatigue and suicidal ideation [7] . Heat shock proteins (HSPs) can be induced by a variety of stressful conditions and are an evolutionarily conserved superfamily comprising a group of structurally unrelated subfamilies, including HSP70, HSP27, HSP90, HSP110, and HSP40 [46]. The serum of BD patients contained increased HSP60 and HSP70 and decreased HSP10 levels, compared to that of healthy controls [47]. Moreover, dysregulation of HSP70, HSP90, and heat shock transcriptional factors 1 and 4 was detected in lymphocytes of BD patients [48]. HSPA12A is a recently identified HSP that has been classified as a distant member of the HSP70 family due to its split ATPase domain [49]. We and others have reported that HSPA12A is highly expressed in the brain [49, 50]. Intriguingly, evidence indicates a potential involvement of HSPA12A in the pathogeneses of psychiatric disorders because HSPA12A expression is reduced in the brains of patients with schizophrenia [18]. Although we have recently demonstrated that HSPA12A encodes a prosurvival pathway against ischemic stroke [20], the causal effects of HSPA12A in psychiatric disorders are completely unknown. Here, we found that acute psychological stress increased HSPA12A expression in the hippocampus but not in the frontal cortex, whereas HSPA12A knockout resulted in mood instability, including anhedonia, low locomotor activity, and antidepressive and anxiety-like behaviors. Taken together, our results provide clear evidence that HSPA12A is a novel psychological regulator and is required for mood stabilization.

The mechanism underlying the development of mood disorder has not been clearly addressed; however, both clinical and experimental studies suggest that it could involve multiple aspects of dysregulation. Among them, impairment of AHN plays an important role in mood disorders development, partially because the adult-born hippocampal neurons are essential for the normal expression of the endocrine and behavioral components of the stress response [9]. For example, AHN was compromised when *negr1* knockout mice developed anxiety- and depression-like behaviors, whereas AHN restoration rescued the mood disorders [8]. A direct role of AHN in mood regulation was also demonstrated by Snyder et al. [9]. After inhibiting AHN via either transgenic or radiation methods, mice show increased food avoidance in a novel environment after acute stress, increased behavioral despair in the forced swim test, and decreased sucrose preference (a measure of anhedonia) [9]. Also, we found that HSPA12A deficiency reduced total dendrite length and dendrite spine density in hippocampal neurons of mice, suggesting a possible involvement of spinogenesis in mood regulation. Indeed, previous studies demonstrated an impaired spinogenesis in mice with behavioral alteration, whereas maintenance of synaptic protein expression ameliorates hyperactivity and anxiety [51–53]. Moreover, neuropsychiatric disorders can lead to abnormal synaptic development or function of hippocampal pyramidal neurons of mice [54]. These findings suggest a complicated, reciprocal causation between dendrite structure and behavior abnormalities. Together, our study revealed impaired AHN, decreased dendritic length and dendritic spines, and downregulated BDNF expression in *Hspa12a*^−^^*/*^^−^ mice that developed mood instability, suggesting an involvement of AHN in the regulatory effect of HSPA12A on mood and behavior.

Studies have demonstrated that AHN can be positively regulated by lactate [11–13]. Lactate is an end product of glycolysis and is also implicated in mood regulation [12, 15, 16]. For example, cerebral lactate levels are altered during episodes of depression and anxiety in BD patients [14]. Additionally, supplement with lactate is effective in treating social avoidance, anxiety, and sleep onset problems that associated with depression in addition to promoting resilience to stress [12, 15, 16]. These findings provide a framework between cerebral lactate, AHN, and mood stability. In this study, we used mouse and primary hippocampal neuron culture models and demonstrated the following results: 1) acute psychological stress increased HSPA12A expression in the hippocampus but not in the frontal cortex, which was accompanied by increased CSF lactate levels and hippocampal glycolysis-related gene expression; 2) HSPA12A knockout resulted in mood instability, impaired AHN, decreased CSF lactate levels, and downregulated hippocampal expression of glycolysis-related genes; 3) HSPA12A is required for maintaining aerobic glycolysis to generate lactate in hippocampal neurons; and 4) chronic and peripheral lactate administration not only increased CSF lactate levels but also rescued mood instability and AHN impairment in *Hspa12a*^−^^*/*^^−^ mice. Taken together, our results provide evidence that HSPA12A controls cerebral lactate homeostasis to modulate AHN and mood stability.

GSK3β is an evolutionarily highly conserved serine/threonine kinase with high expression levels in the brain. GSK3β activity is negatively regulated by Ser9 phosphorylation. Decreased GSK3β phosphorylation, which causes increased GSK3β activity in specific cellular locations, pathways, and circuits, promotes susceptibility to mood disorders, and GSK3β inhibition is therapeutic for mood disorders [55–57]. Lithium, a GSK3β inhibitor, is a first-line mood-stabilizing treatment for BD [57]. However, whether GSK3β plays roles in glycolysis regulation is poorly known. Interestingly, using both mouse and primary neuron culture models, we found the following results: 1) when *Hspa12a*^−^^*/*^^−^ mice developed mood instability and AHN impairment, GSK3β phosphorylation levels were also decreased in the hippocampus; 2) HSPA12A overexpression increased GSK3β phosphorylation in primary hippocampal neurons; 3) the GSK3β activator DIF3 reversed the HSPA12A-promoted lactate production and glycolysis-related gene expression in hippocampal neurons; and 4) the GSK3β inhibitor lithium improved lactate levels in CSF, rescued AHN impairment and mood instability in *Hspa12a*^−^^*/*^^−^ mice. Our findings suggest that GSK3β inhibition mediates the regulatory effects of HSPA12A on cerebral lactate levels to modulate appropriate AHN and mood status.

In conclusion, this study demonstrated that HSPA12A is required for maintaining AHN and mood stabilization by sustaining cerebral lactate homeostasis which could be mediated by inhibiting GSK3β in hippocampal neurons. Thus, HSPA12A is considered as a new regulator of AHN and mood stabilization that may have therapeutic potential for mood disorders.

### Supplementary information

Supplemental video

Supplemental results

Supplemental material

Supplemental tables

## References

[1] Ward J, Tunbridge E, Sandor C, Lyall L, Ferguson A, Strawbridge R (2020). The genomic basis of mood instability: identification of 46 loci in 363,705 UK Biobank participants, genetic correlation with psychiatric disorders, and association with gene expression and function. Mol Psych.

[2] Ward J, Strawbridge R, Bailey M, Graham N, Ferguson A, Lyall D (2017). Genome-wide analysis in UK Biobank identifies four loci associated with mood instability and genetic correlation with major depressive disorder, anxiety disorder and schizophrenia. Transl Psych.

[3] Miklowitz D, Weintraub M, Singh M, Walshaw P, Merranko J, Birmaher B (2022). Mood Instability in Youth at High Risk for Bipolar Disorder. J Am Acad Child Adolesc Psych.

[4] Stanislaus S, Faurholt-Jepsen M, Vinberg M, Coello K, Kjærstad H, Melbye S (2020). Mood instability in patients with newly diagnosed bipolar disorder, unaffected relatives, and healthy control individuals measured daily using smartphones. J Affect Disord.

[5] Young AH (2022). The psychopharmacology of mood disorders. J Psychopharmacol.

[6] Crowe M (2017). Recovery and mood disorders. J Psychiatr Ment Health Nurs.

[7] Rakofsky J, Rapaport M (2018). Mood Disorders. Contin (Minneap Minn).

[8] Noh K, Lee H, Choi T, Joo Y, Kim S, Kim H (2019). Negr1 controls adult hippocampal neurogenesis and affective behaviors. Mol Psych.

[9] Snyder J, Soumier A, Brewer M, Pickel J, Cameron H (2011). Adult hippocampal neurogenesis buffers stress responses and depressive behaviour. Nature..

[10] Carli M, Aringhieri S, Kolachalam S, Longoni B, Grenno G, Rossi M (2021). Is Adult Hippocampal Neurogenesis Really Relevant for the Treatment of Psychiatric Disorders?. Curr Neuropharmacol.

[11] Wang J, Cui Y, Yu Z, Wang W, Cheng X, Ji W (2019). Brain Endothelial Cells Maintain Lactate Homeostasis and Control Adult Hippocampal Neurogenesis. Cell Stem Cell.

[12] Carrard A, Cassé F, Carron C, Burlet-Godinot S, Toni N, Magistretti P (2021). Role of adult hippocampal neurogenesis in the antidepressant actions of lactate. Mol Psych.

[13] Nicola R, Okun E (2021). Adult Hippocampal Neurogenesis: One Lactate to Rule Them All. Neuromolecular Med.

[14] Dogan A, Yuksel C, Du F, Chouinard V, Öngür D (2018). Brain lactate and pH in schizophrenia and bipolar disorder: a systematic review of findings from magnetic resonance studies. Neuropsychopharmacology..

[15] Murack M, Messier C (2019). The impact of lactic acid and medium chain triglyceride on blood glucose, lactate and diurnal motor activity: A re-examination of a treatment of major depression using lactic acid. Physiol Behav.

[16] Karnib N, El-Ghandour R, El Hayek L, Nasrallah P, Khalifeh M, Barmo N (2019). Lactate is an antidepressant that mediates resilience to stress by modulating the hippocampal levels and activity of histone deacetylases. Neuropsychopharmacology..

[17] Han Z, Truong QA, Park S, Breslow JL (2003). Two Hsp70 family members expressed in atherosclerotic lesions. Proc Natl Acad Sci USA.

[18] Pongrac JL, Middleton FA, Peng L, Lewis DA, Levitt P, Mirnics K (2004). Heat shock protein 12A shows reduced expression in the prefrontal cortex of subjects with schizophrenia. Biol Psych.

[19] Kong Q, Li N, Cheng H, Zhang X, Cao X, Qi T (2019). HSPA12A Is a Novel Player in Nonalcoholic Steatohepatitis via Promoting Nuclear PKM2-Mediated M1 Macrophage Polarization. Diabetes..

[20] Mao Y, Kong Q, Li R, Zhang X, Gui Y, Li Y (2018). Heat shock protein A12A encodes a novel prosurvival pathway during ischaemic stroke. Biochim Biophys Acta Mol Basis Dis.

[21] Min X, Zhang X, Li Y, Cao X, Cheng H, Li Y (2020). HSPA12A unstabilizes CD147 to inhibit lactate export and migration in human renal cell carcinoma. Theranostics..

[22] Mao Y, Kong Q, Li R, Zhang X, Gui Y, Li Y (2018). Heat shock protein A12A encodes a novel prosurvival pathway during ischaemic stroke. Biochim Biophys Acta Mol Basis Dis.

[23] Avital A, Richter-Levin G, Leschiner S, Spanier I, Veenman L, Weizman A (2001). Acute and repeated swim stress effects on peripheral benzodiazepine receptors in the rat hippocampus, adrenal, and kidney. Neuropsychopharmacology..

[24] Browne CA, Hanke J, Rose C, Walsh I, Foley T, Clarke G (2014). Effect of acute swim stress on plasma corticosterone and brain monoamine levels in bidirectionally selected DxH recombinant inbred mouse strains differing in fear recall and extinction. Stress..

[25] Li J, Sha L, Xu Q (2020). An early increase in glutamate is critical for the development of depression-like behavior in a chronic restraint stress (CRS) model. Brain Res Bull.

[26] Zhang W, Liu W, He Y, You W, Zhang J, Xu H (2019). Chronic Stress Causes Projection-Specific Adaptation of Amygdala Neurons via Small-Conductance Calcium-Activated Potassium Channel Downregulation. Biol Psych.

[27] Li Y, Jia Y, Wang D, Zhuang X, Li Y, Guo C (2021). Programmed cell death 4 as an endogenous suppressor of BDNF translation is involved in stress-induced depression. Mol Psych.

[28] Yu X, Ba W, Zhao G, Ma Y, Harding E, Yin L (2021). Dysfunction of ventral tegmental area GABA neurons causes mania-like behavior. Mol Psych.

[29] Spadaro PA, Flavell CR, Widagdo J, Ratnu VS, Troup M, Ragan C (2015). Long Noncoding RNA-Directed Epigenetic Regulation of Gene Expression Is Associated with Anxiety-like Behavior in Mice. Biol Psych.

[30] Ito W, Chehab M, Thakur S, Li J, Morozov A (2011). BDNF-restricted knockout mice as an animal model for aggression. Genes Brain Behav.

[31] Bailey KR, Crawley JN Anxiety-Related Behaviors in Mice. In: Buccafusco JJ, editor. Methods of Behavior Analysis in Neuroscience. 2nd ed. Boca Raton (FL); 2009.

[32] Papaleo F, Silverman JL, Aney J, Tian Q, Barkan CL, Chadman KK (2011). Working memory deficits, increased anxiety-like traits, and seizure susceptibility in BDNF overexpressing mice. Learn Mem.

[33] Amar M, Pramod A, Yu N, Herrera V, Qiu L, Moran-Losada P (2021). Autism-linked Cullin3 germline haploinsufficiency impacts cytoskeletal dynamics and cortical neurogenesis through RhoA signaling. Mol Psych.

[34] Carrard A, Elsayed M, Margineanu M, Boury-Jamot B, Fragniere L, Meylan EM (2018). Peripheral administration of lactate produces antidepressant-like effects. Mol Psych.

[35] Roybal K, Theobold D, Graham A, DiNieri JA, Russo SJ, Krishnan V (2007). Mania-like behavior induced by disruption of CLOCK. Proc Natl Acad Sci USA.

[36] Takahashi-Yanaga F, Taba Y, Miwa Y, Kubohara Y, Watanabe Y, Hirata M (2003). Dictyostelium differentiation-inducing factor-3 activates glycogen synthase kinase-3beta and degrades cyclin D1 in mammalian cells. J Biol Chem.

[37] Takahashi-Yanaga F, Mori J, Matsuzaki E, Watanabe Y, Hirata M, Miwa Y (2006). Involvement of GSK-3beta and DYRK1B in differentiation-inducing factor-3-induced phosphorylation of cyclin D1 in HeLa cells. J Biol Chem.

[38] Sepehr A, Mohseni S (2009). Lactate damages primary hippocampal neurons in vitro. Cell Biol Int.

[39] Lim NK, Moestrup V, Zhang X, Wang WA, Moller A, Huang FD (2018). An Improved Method for Collection of Cerebrospinal Fluid from Anesthetized Mice. J Vis Exp.

[40] Kong Q, Dai L, Wang Y, Zhang X, Li C, Jiang S (2016). HSPA12B Attenuated Acute Myocardial Ischemia/reperfusion Injury via Maintaining Endothelial Integrity in a PI3K/Akt/mTOR-dependent Mechanism. Sci Rep.

[41] Liu J, Du S, Kong Q, Zhang X, Jiang S, Cao X (2020). HSPA12A attenuates lipopolysaccharide-induced liver injury through inhibiting caspase-11-mediated hepatocyte pyroptosis via PGC-1alpha-dependent acyloxyacyl hydrolase expression. Cell Death Differ.

[42] Chen YF, Chen ZX, Wang RH, Shi YW, Xue L, Wang XG (2019). Knockdown of CLC-3 in the hippocampal CA1 impairs contextual fear memory. Prog Neuropsychopharmacol Biol Psych.

[43] Mohammad H, Marchisella F, Ortega-Martinez S, Hollos P, Eerola K, Komulainen E (2018). JNK1 controls adult hippocampal neurogenesis and imposes cell-autonomous control of anxiety behaviour from the neurogenic niche. Mol Psych.

[44] Millan M, Rivet J, Gobert A (2016). The frontal cortex as a network hub controlling mood and cognition: Probing its neurochemical substrates for improved therapy of psychiatric and neurological disorders. J Psychopharmacol.

[45] Leschik J, Gentile A, Cicek C, Péron S, Tevosian M, Beer A (2022). Brain-derived neurotrophic factor expression in serotonergic neurons improves stress resilience and promotes adult hippocampal neurogenesis. Prog Neurobiol.

[46] Vos MJ, Hageman J, Carra S, Kampinga HH (2008). Structural and functional diversities between members of the human HSPB, HSPH, HSPA, and DNAJ chaperone families. Biochemistry..

[47] Cheng Y, Li Z, He S, Tian Y, He F, Li W (2018). Elevated heat shock proteins in bipolar disorder patients with hypothalamic pituitary adrenal axis dysfunction. Medicine..

[48] Bei E, Salpeas V, Alevizos B, Anagnostara C, Pappa D, Moutsatsou P (2013). Pattern of heat shock factor and heat shock protein expression in lymphocytes of bipolar patients: increased HSP70-glucocorticoid receptor heterocomplex. J Psychiatr Res.

[49] Han Z, Truong Q, Park S, Breslow J (2003). Two Hsp70 family members expressed in atherosclerotic lesions. Proc Natl Acad Sci USA.

[50] Zhang X, Chen X, Qi T, Kong Q, Cheng H, Cao X (2019). HSPA12A is required for adipocyte differentiation and diet-induced obesity through a positive feedback regulation with PPARgamma. Cell Death Differ.

[51] Shih PY, Hsieh BY, Lin MH, Huang TN, Tsai CY, Pong WL (2020). CTTNBP2 Controls Synaptic Expression of Zinc-Related Autism-Associated Proteins and Regulates Synapse Formation and Autism-like Behaviors. Cell Rep.

[52] Chenani A, Weston G, Ulivi AF, Castello-Waldow TP, Huettl RE, Chen A (2022). Repeated stress exposure leads to structural synaptic instability prior to disorganization of hippocampal coding and impairments in learning. Transl Psych.

[53] Uchida S, Hara K, Kobayashi A, Fujimoto M, Otsuki K, Yamagata H (2011). Impaired hippocampal spinogenesis and neurogenesis and altered affective behavior in mice lacking heat shock factor 1. Proc Natl Acad Sci USA.

[54] Han K, Holder JL, Schaaf CP, Lu H, Chen H, Kang H (2013). SHANK3 overexpression causes manic-like behaviour with unique pharmacogenetic properties. Nature..

[55] Jope RS (2011). Glycogen synthase kinase-3 in the etiology and treatment of mood disorders. Front Mol Neurosci.

[56] Prickaerts J, Moechars D, Cryns K, Lenaerts I, van Craenendonck H, Goris I (2006). Transgenic mice overexpressing glycogen synthase kinase 3beta: a putative model of hyperactivity and mania. J Neurosci.

[57] Köhler-Forsberg O, Rohde C, Nierenberg A, Østergaard S (2022). Association of Lithium Treatment With the Risk of Osteoporosis in Patients With Bipolar Disorder. JAMA Psych.

