# Supplementary material for: HSPA12A controls cerebral lactate homeostasis to maintain hippocampal neurogenesis and mood stabilization
Source: Transl Psychiatry. 2023 Aug 14;13:280. doi: 10.1038/s41398-023-02573-5 (PMC10425330; doi:10.1038/s41398-023-02573-5)
Supplement: Supplementary file 2 — Supplemental results [file 41398_2023_2573_MOESM2_ESM.pdf]

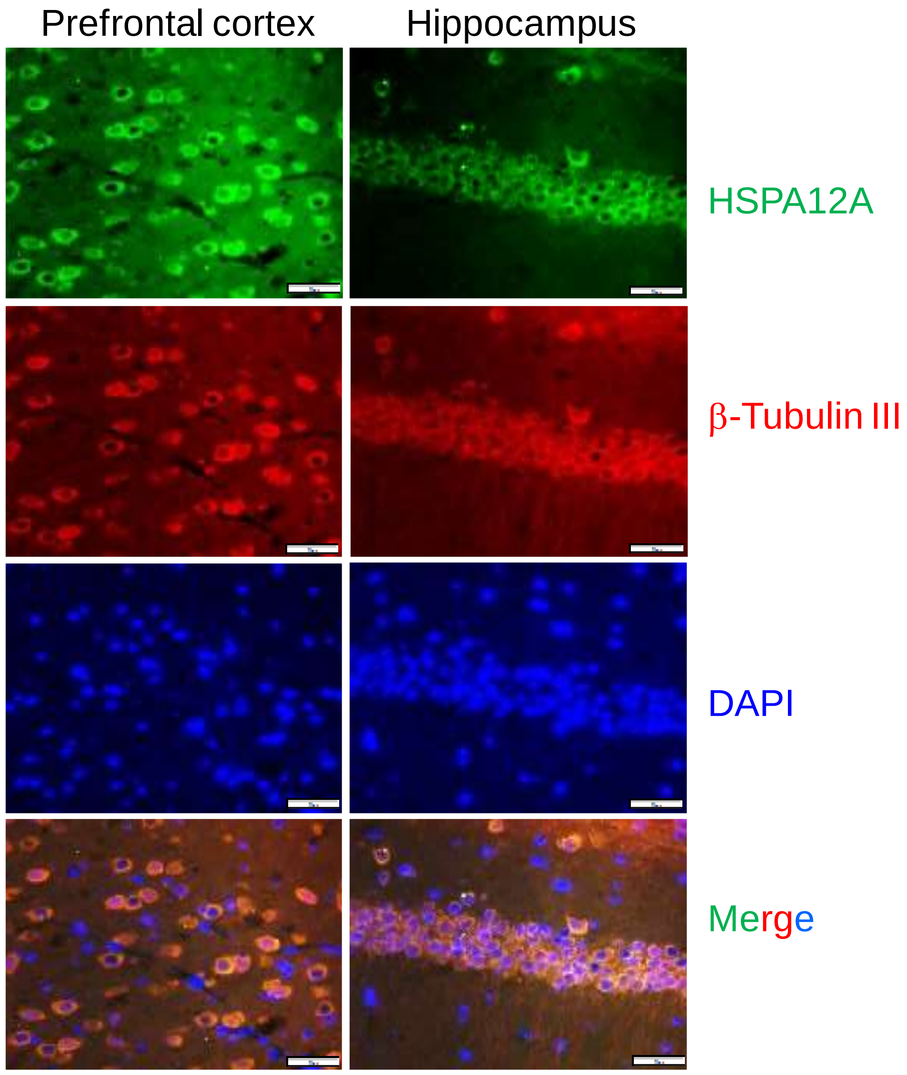
Prefrontal cortex
Hippocampus
HSPA12A
β-Tubulin III
DAPI
Merge

**Figure S1**

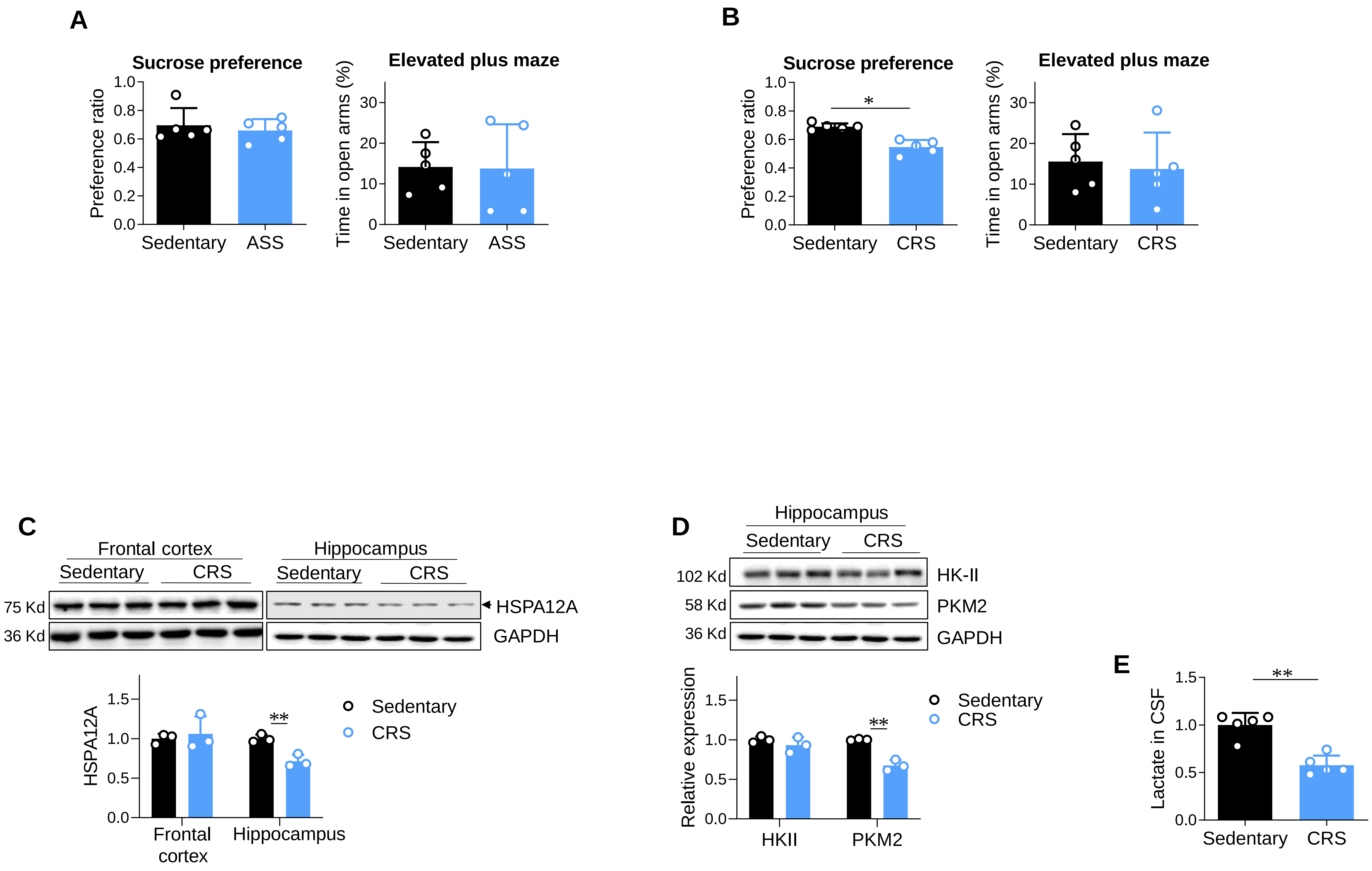

A
Sucrose preference
Preference ratio
1.0
0.8
0.6
0.4
0.2
0.0
Sedentary
ASS
Elevated plus maze
Time in open arms (%)
30
20
10
0
Sedentary
ASS
B
Sucrose preference
Preference ratio
*
Sedentary
CRS
Elevated plus maze
Time in open arms (%)
Sedentary
CRS
C
Frontal cortex
Hippocampus
Sedentary
CRS
75 Kd
36 Kd
HSPA12A
GAPDH
HSPA12A
1.5
1.0
0.5
0.0
**
Frontal cortex
Hippocampus
Sedentary
CRS
D
Hippocampus
Sedentary
CRS
102 Kd
58 Kd
36 Kd
HK-II
PKM2
GAPDH
Relative expression
1.5
1.0
0.5
0.0
**
HKII
PKM2
Sedentary
CRS
E
Lactate in CSF
1.5
1.0
0.5
0.0
**
Sedentary
CRS


**Figure S2**

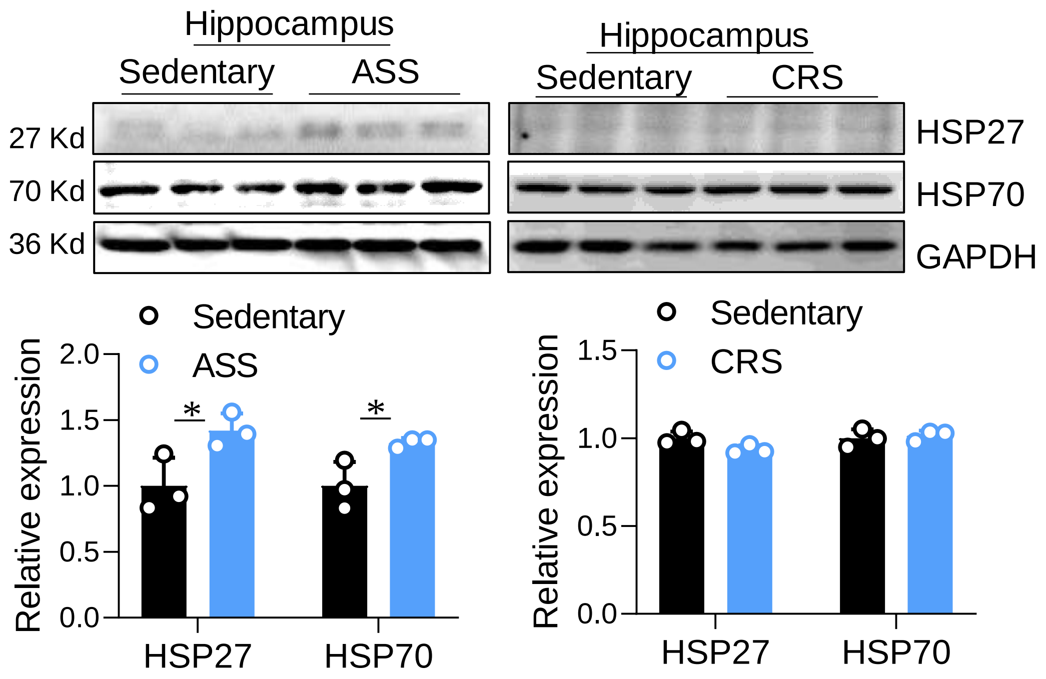
Hippocampus
Sedentary
ASS
27 Kd
70 Kd
36 Kd
Hippocampus
Sedentary
CRS
HSP27
HSP70
GAPDH
Sedentary
ASS
Relative expression
2.0
1.5
1.0
0.5
0.0
*
*
HSP27
HSP70
Sedentary
CRS
Relative expression
1.5
1.0
0.5
0.0
HSP27
HSP70

**Figure S3**

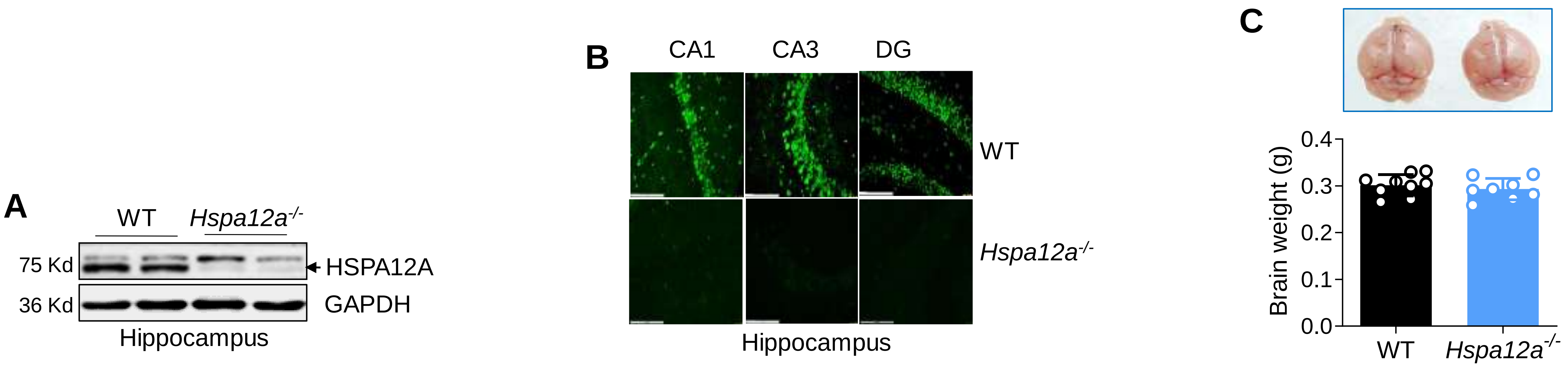
A
WT
Hspa12a-/-
75 Kd
36 Kd
HSPA12A
GAPDH
Hippocampus
B
CA1
CA3
DG
WT
Hspa12a-/-
Hippocampus
C
Brain weight (g)
0.4
0.3
0.2
0.1
0.0
WT
Hspa12a-/-

**Figure S4**

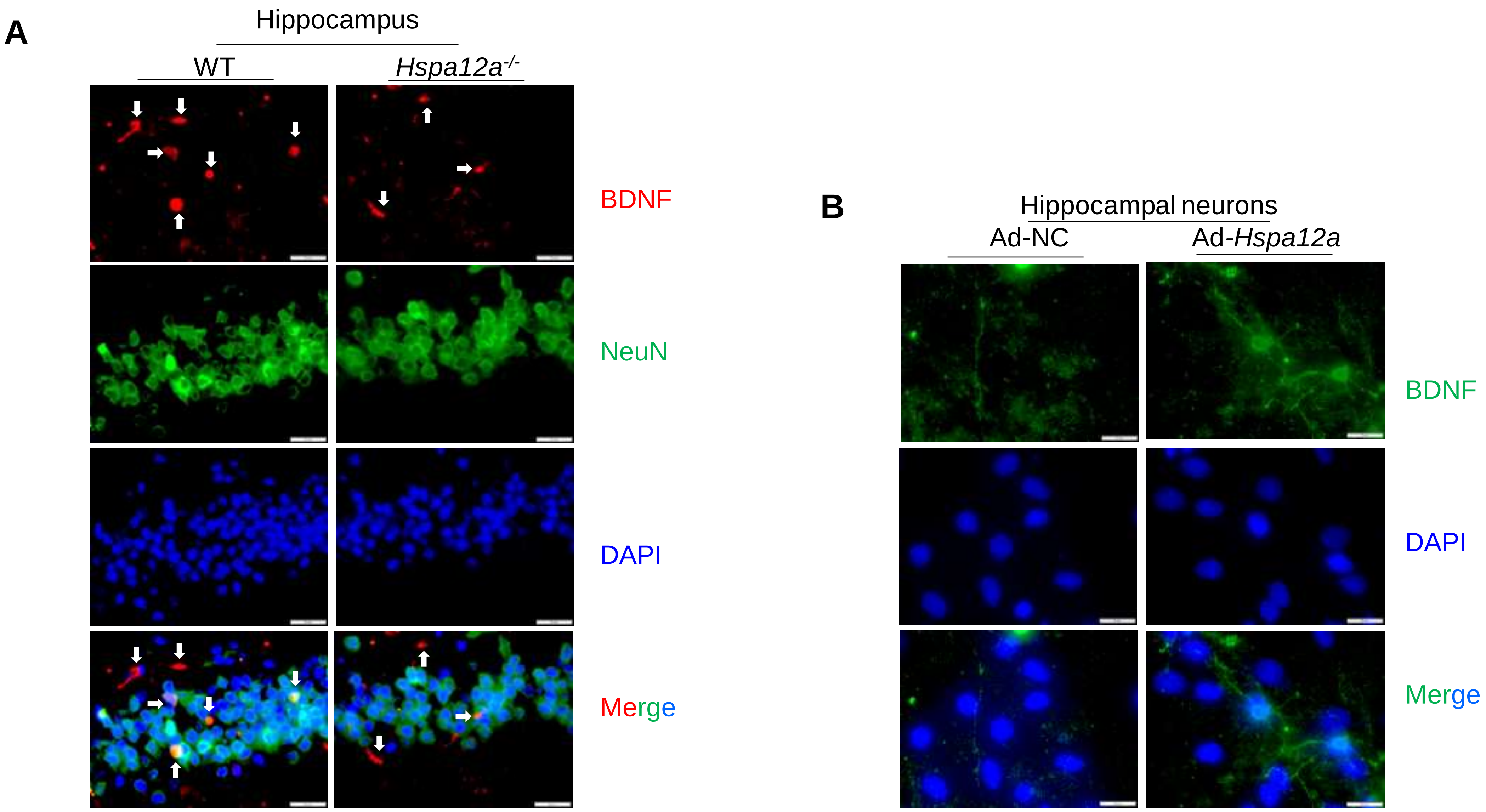
A
Hippocampus
WT
Hspa12a-/-
BDNF
NeuN
DAPI
Merge
B
Hippocampal neurons
Ad-NC
Ad-Hspa12a
BDNF
DAPI
Merge

**Figure S5**

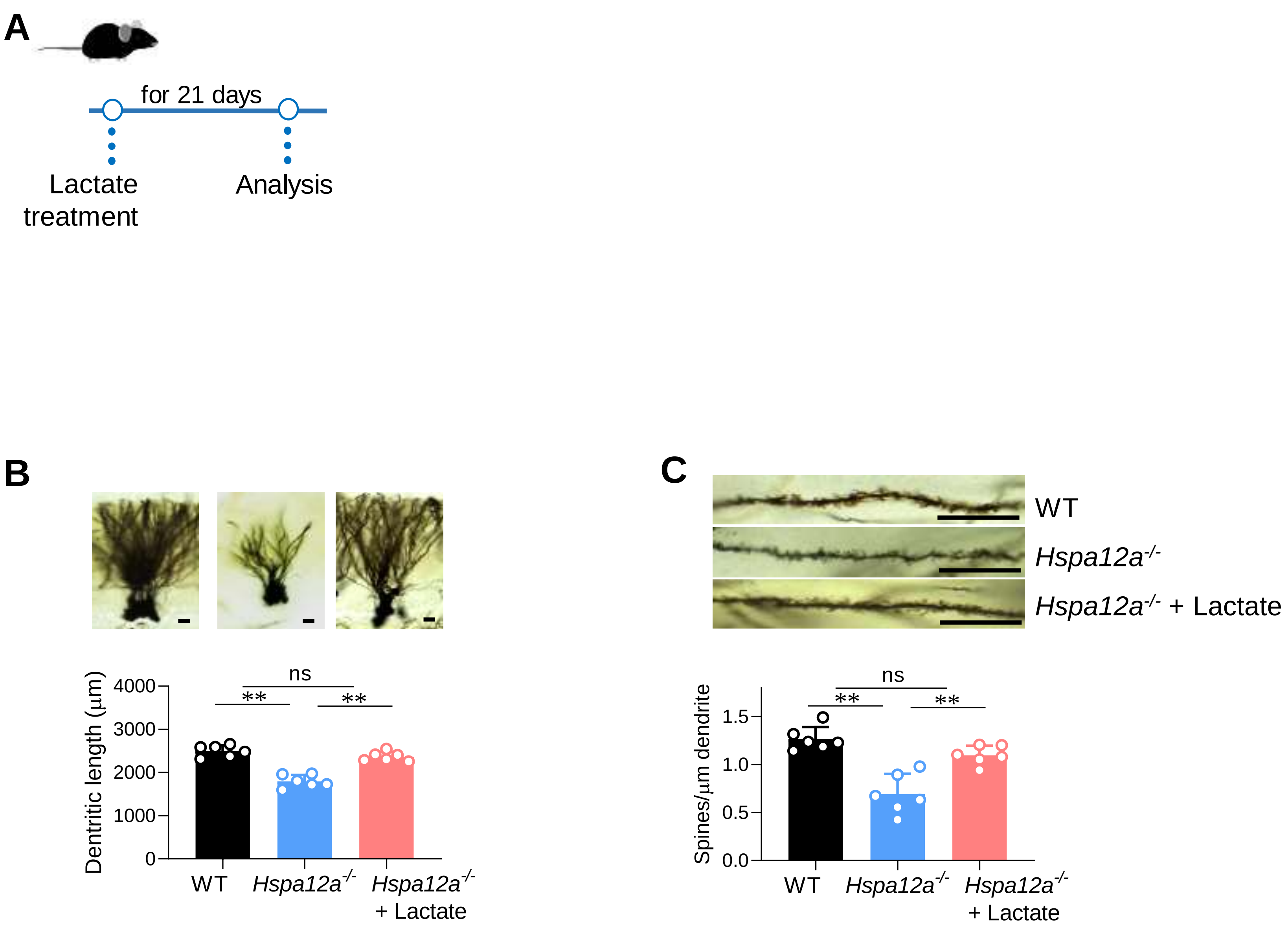
A
for 21 days
Lactate treatment
Analysis
B
4000
3000
2000
1000
0
Dentritic length (μm)
ns
**
**
WT
Hspa12a-/-
Hspa12a-/- + Lactate
C
WT
Hspa12a-/-
Hspa12a-/- + Lactate
1.5
1.0
0.5
0.0
Spines/μm dendrite
ns
**
**
WT
Hspa12a-/-
Hspa12a-/- + Lactate

**Figure S6**

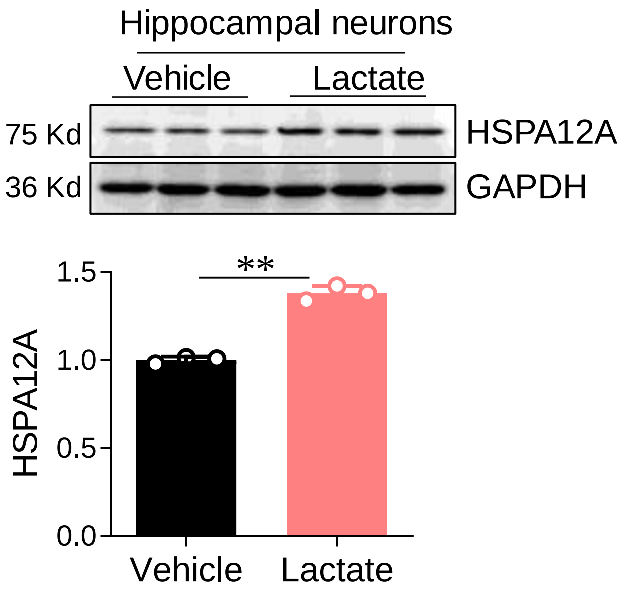
Hippocampal neurons
Vehicle
Lactate
75 Kd
36 Kd
HSPA12A
GAPDH
**
1.5
1.0
0.5
0.0
HSPA12A
Vehicle
Lactate

**Figure S7**

A
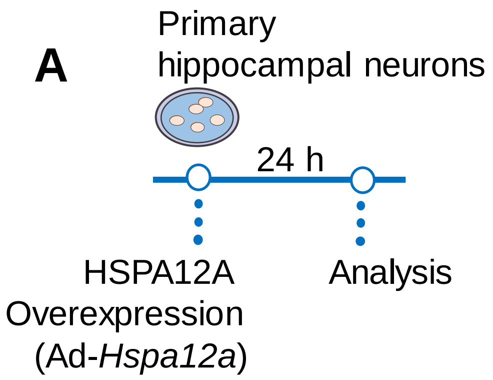
Primary
hippocampal neurons
24 h
HSPA12A
Overexpression
(Ad-Hspa12a)
Analysis

B
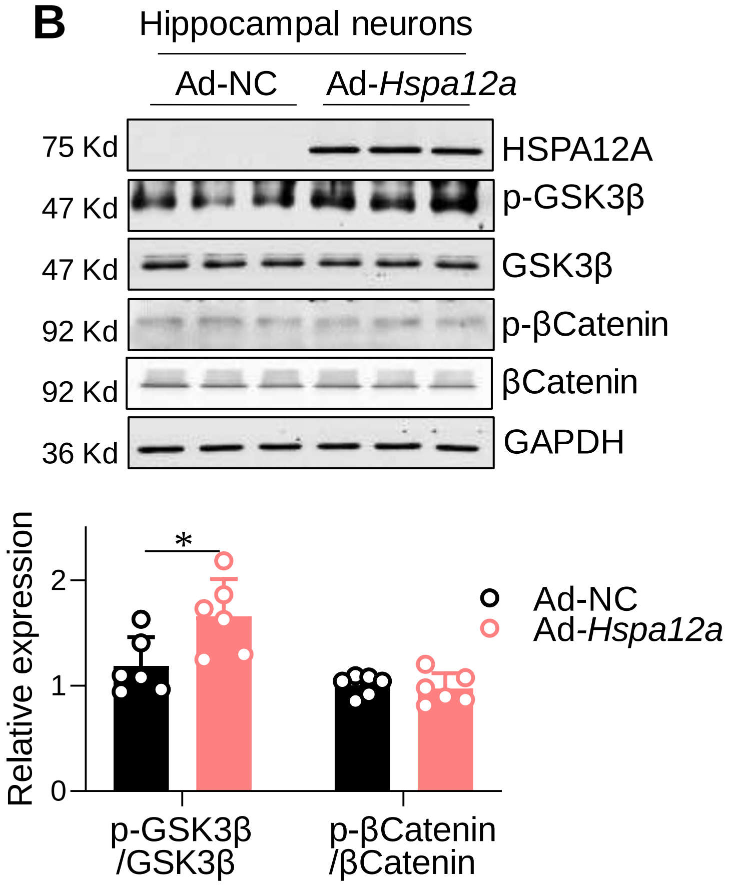
Hippocampal neurons
Ad-NC
Ad-Hspa12a
75 Kd
HSPA12A
47 Kd
p-GSK3β
47 Kd
GSK3β
92 Kd
p-βCatenin
92 Kd
βCatenin
36 Kd
GAPDH
Relative expression
*
2
1
0
Ad-NC
Ad-Hspa12a
p-GSK3β
/GSK3β
p-βCatenin
/βCatenin

**Figure S8**

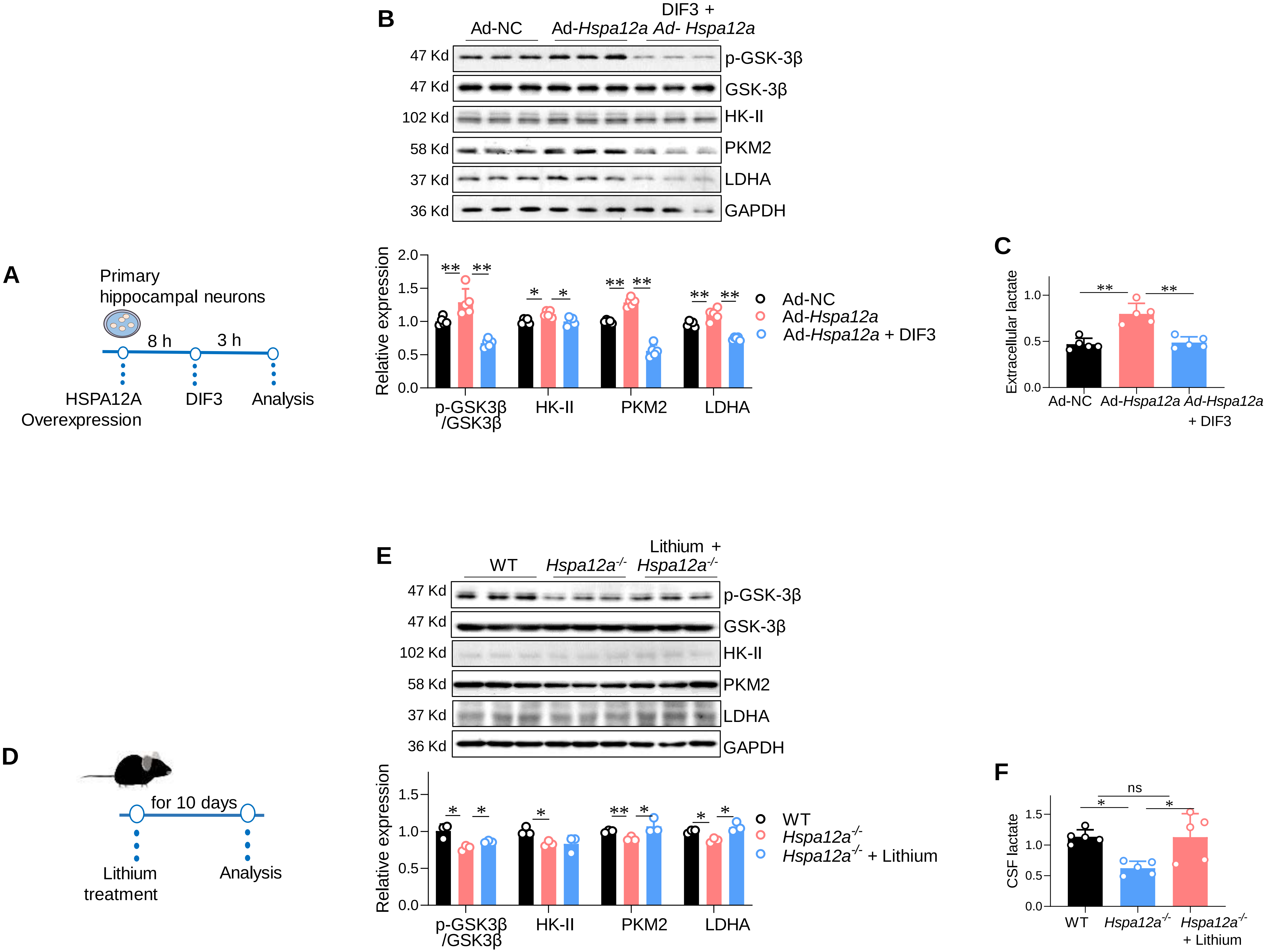
A
Primary
hippocampal neurons
8 h
3 h
HSPA12A
Overexpression
DIF3
Analysis
B
Ad-NC
Ad-Hspa12a
DIF3 +
Ad- Hspa12a
47 Kd
p-GSK-3β
47 Kd
GSK-3β
102 Kd
HK-II
58 Kd
PKM2
37 Kd
LDHA
36 Kd
GAPDH
Relative expression
p-GSK3β
/GSK3β
HK-II
PKM2
LDHA
Ad-NC
Ad-Hspa12a
Ad-Hspa12a + DIF3
C
Extracellular lactate
Ad-NC
Ad-Hspa12a
Ad-Hspa12a
+ DIF3
D
for 10 days
Lithium
treatment
Analysis
E
WT
Hspa12a-/-
Lithium +
Hspa12a-/-
47 Kd
p-GSK-3β
47 Kd
GSK-3β
102 Kd
HK-II
58 Kd
PKM2
37 Kd
LDHA
36 Kd
GAPDH
Relative expression
p-GSK3β
/GSK3β
HK-II
PKM2
LDHA
WT
Hspa12a-/-
Hspa12a-/- + Lithium
F
CSF lactate
ns
WT
Hspa12a-/-
Hspa12a-/-
+ Lithium

**Figure S9**

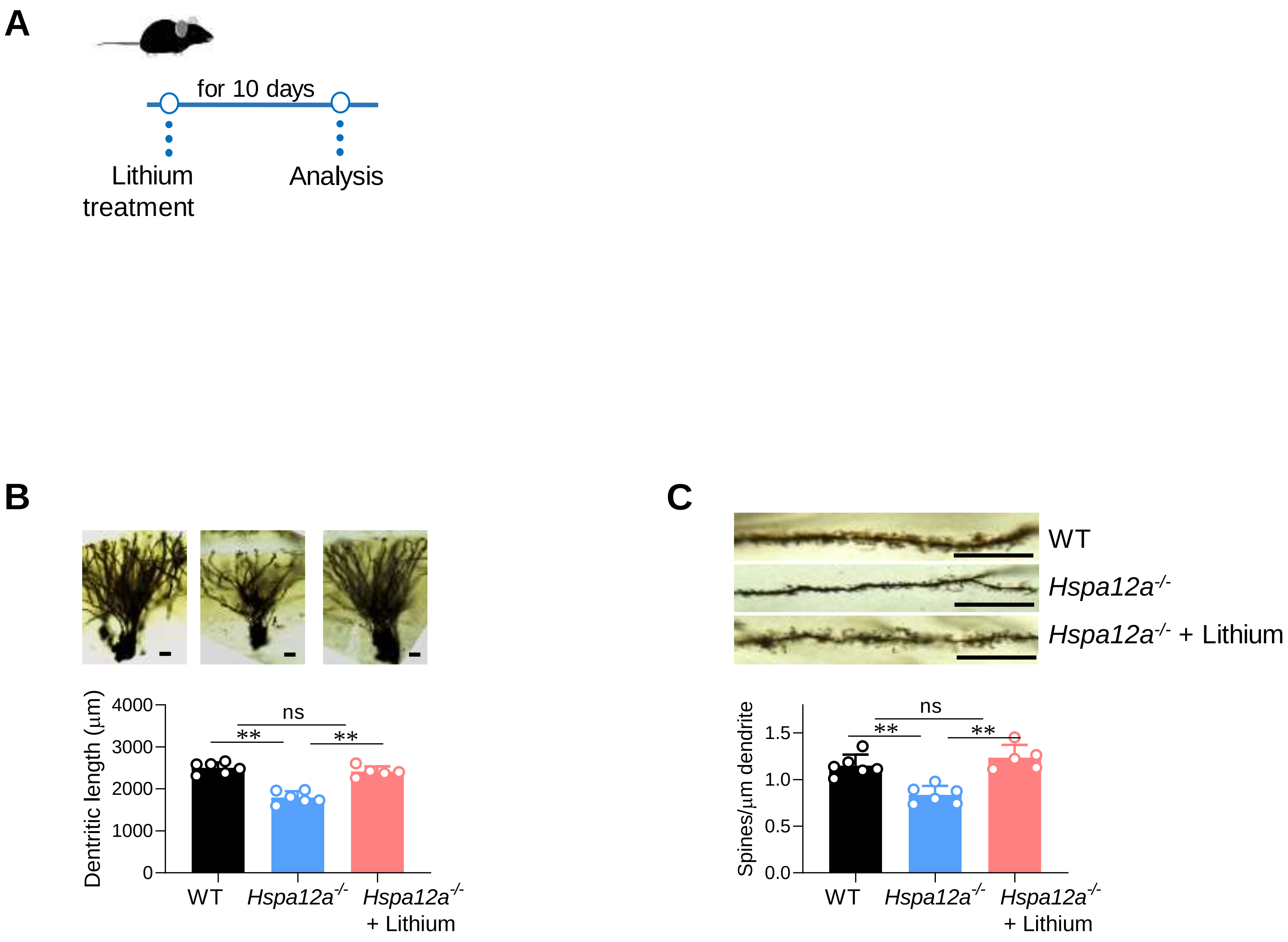
A
for 10 days
Lithium treatment
Analysis
B
Dentritic length (μm)
4000
3000
2000
1000
0
ns
**
**
WT
Hspa12a-/-
Hspa12a-/- + Lithium
C
WT
Hspa12a-/-
Hspa12a-/- + Lithium
Spines/μm dendrite
1.5
1.0
0.5
0.0
ns
**
**
WT
Hspa12a-/-
Hspa12a-/- + Lithium

**Figure S10**

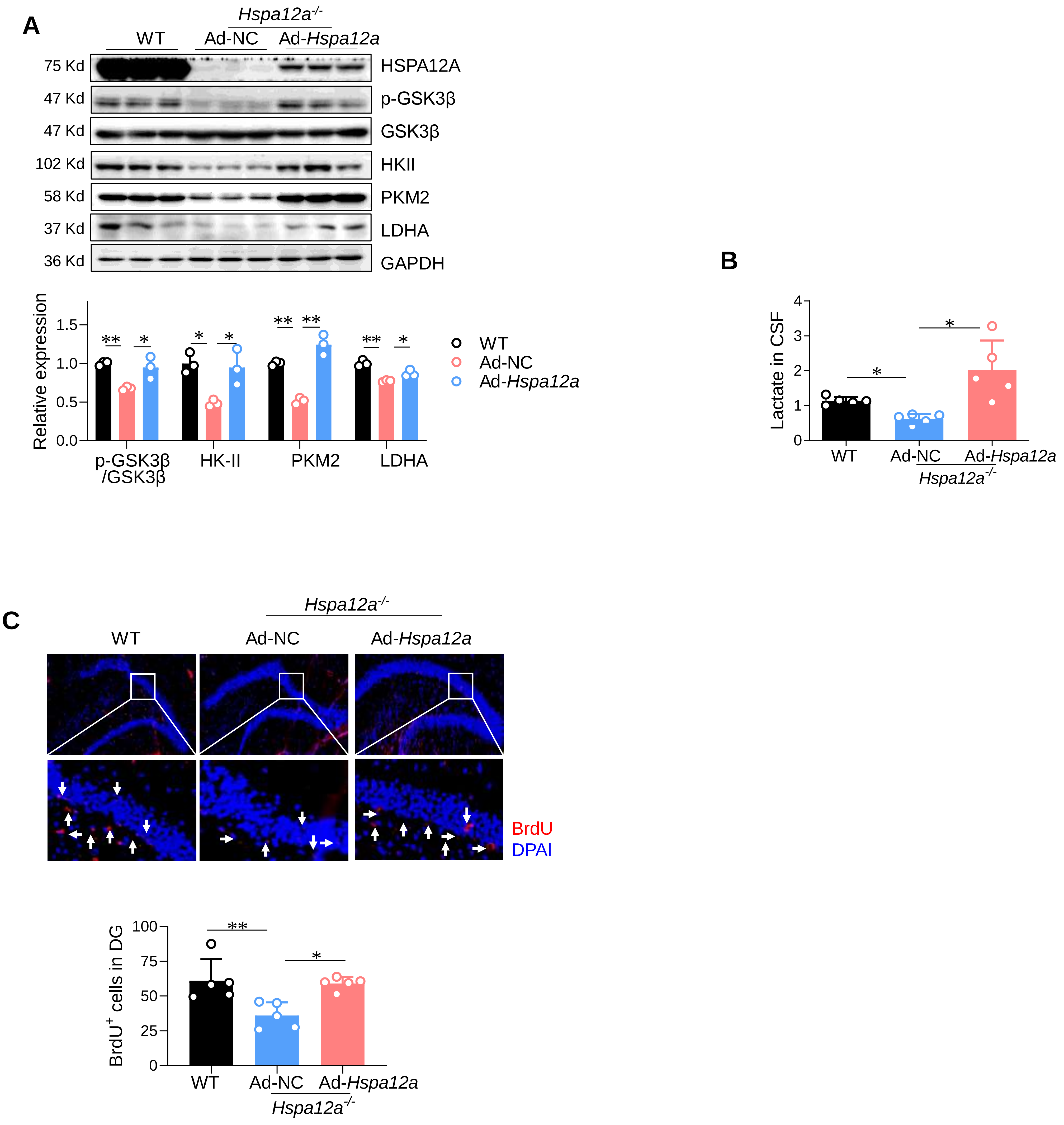
A
Hspa12a-/-
WT
Ad-NC
Ad-Hspa12a
75 Kd
47 Kd
47 Kd
102 Kd
58 Kd
37 Kd
36 Kd
HSPA12A
p-GSK3β
GSK3β
HKII
PKM2
LDHA
GAPDH
Relative expression
1.5
1.0
0.5
0.0
**
*
*
*
**
**
**
*
p-GSK3β
/GSK3β
HK-II
PKM2
LDHA
WT
Ad-NC
Ad-Hspa12a
B
Lactate in CSF
4
3
2
1
0
*
*
WT
Ad-NC
Ad-Hspa12a
Hspa12a-/-
C
Hspa12a-/-
WT
Ad-NC
Ad-Hspa12a
BrdU
DPAI
BrdU+ cells in DG
100
75
50
25
0
**
*
WT
Ad-NC
Ad-Hspa12a
Hspa12a-/-

**Figure S11**

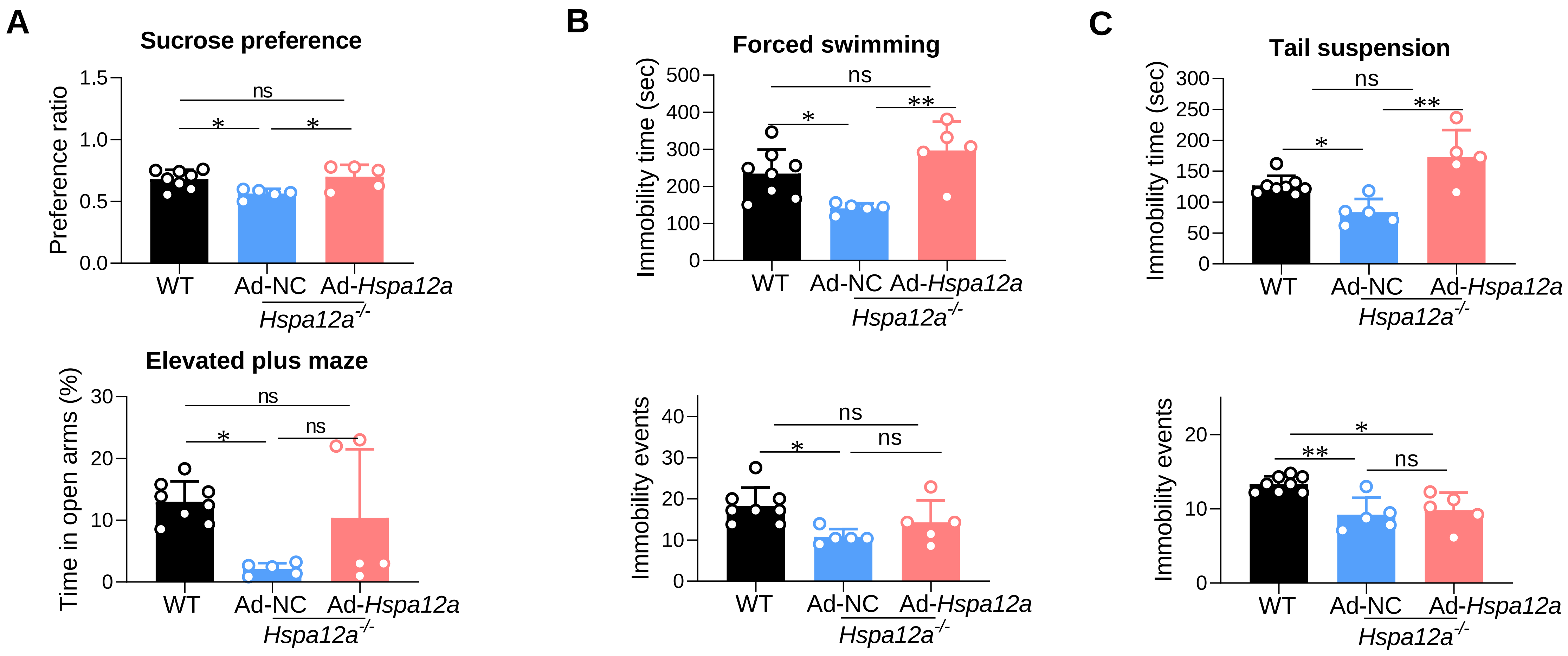
A
Sucrose preference
Preference ratio
1.5
1.0
0.5
0.0
ns
*
*
WT
Ad-NC
Ad-*Hspa12a*
*Hspa12a*-/-
Elevated plus maze
Time in open arms (%)
30
20
10
0
ns
*
ns
B
Forced swimming
Immobility time (sec)
500
400
300
200
100
0
ns
*
**
Immobility events
40
30
20
10
0
ns
*
ns
C
Tail suspension
Immobility time (sec)
300
250
200
150
100
50
0
ns
*
**
Immobility events
20
10
0
*
**
ns

**Figure S12**
