# Supplementary material for: HSPA12A controls cerebral lactate homeostasis to maintain hippocampal neurogenesis and mood stabilization
Source: Transl Psychiatry. 2023 Aug 14;13:280. doi: 10.1038/s41398-023-02573-5 (PMC10425330; doi:10.1038/s41398-023-02573-5)
Supplement: Supplementary file 3 — Supplemental material [file 41398_2023_2573_MOESM3_ESM.pdf]

Supplemental Materials

Figure Legends

Figure S1. HSPA12A expressed in neurons of hippocampus and cortex.

Brain tissues from adult C57BL/6 were processed for immunostaining against HSPA12A and β-Tubulin III. DAPI was used to counter staining the nuclei. n = 5/group. Scale bar = 100 µm.

Figure S2. Effects of acute and chronic mood stresses on behaviors and glycolytic activity of hippocampus of mice.

A-B. Behavioral tests. n = 5/group.

C. HSPA12A expression. After chronic restraint stress (CRS), HSPA12A expression was examined in frontal cortex and hippocampus using immunoblotting. n = 3/group

D. Glycolysis-related gene expression. After CRS, the indicated gene expression was examined in hippocampus using immunoblotting. n = 3/group

E. Lactate levels in cerebrospinal fluid (CSF). After CRS, CSF was collected for measuring lactate levels. n = 5/group

Data are mean ± SD, ** $P < 0.01$ and * $P < 0.05$ by Student's two-tailed unpaired *t* test.

Figure S3. Effects of acute and chronic mood stresses on heat shock protein expression in hippocampus of mice.

After ASS or CRS, the indicated gene expression was examined in hippocampus using immunoblotting. Data are mean ± SD, * $P < 0.05$ by Student's two-tailed unpaired *t* test. n = 3/group.

Figure S4. HSPA12A expression was deleted in hippocampus of *Hspa12a*$^{-/-}$ mice.

A-B. HSPA12A expression in hippocampus. Expression of hippocampal HSPA12A was examined by immunoblotting **(A)** and immunofluorescence staining **(B)**. Note that HSPA12A expression was absent in hippocampus of *Hspa12a*$^{-/-}$ mice. n = 6/group. Scale bar = 100 μm

C. Brain morphology and brain weight. Brains from Adult (8-week-old) WT and *Hspa12a*$^{-/-}$ mice were photographed and weighted. n = 9 for WT group and n = 8 for *Hspa12a*$^{-/-}$ group.

Data are mean ± SD, ** $P < 0.01$ and * $P < 0.05$ by Student's two-tailed unpaired *t* test.

Figure S5. Immunostaining for BDNF.

A. BDNF staining in hippocampus of mice. Brain tissues from WT and *Hspa12a*$^{-/-}$ mice were processed for cryo-sectioning and immunostaining against BDNF and NeuN. DAPI was used to counter staining the nuclei. n = 5/group. Scale bar = 100

μm.

B. BDNF staining in hippocampal neurons. After overexpression of HSPA12A for 24 h, neurons were processed for immunostaining against BDNF. DAPI was used to counter staining the nuclei. n = 5/group. Scale bar = 100 μm.

Figure S6. Effects of lactate treatment on dendrite length and spinogenesis of hippocampal neurons of HSPA12A knockout mice.

A. Experimental protocol. After lactate treatment for 21 days, the following analyses were performed.

B. Neuronal dendrite length. Neuronal dendrite length in dentate gurus was analyzed using Golgi staining. n = 6/group. Scale bar = 50 μm.

C. Neuronal dendrite spine density. Neuronal spine density of dendrite in dentate gurus was analyzed using Golgi staining. n = 6/group. Scale bar = 20 μm.

Data are mean ± SD, ** $P < 0.01$ by one-way ANOVA followed by post-hoc test. ns, no significant difference.

Figure S7. Effects of lactate treatment on HSPA12A expression in hippocampal neurons.

After lactate treatment for 24 h, primary neurons were collected for analyzing HSPA12A expression using immunoblotting. Data are mean ± SD, ** $P < 0.01$ by Student's two-tailed unpaired *t* test.

Figure S8. HSPA12A inhibited GSK-3β in hippocampal neurons.

A. **Experimental protocol of primary hippocampal neurons.**

B. **Immunoblotting in hippocampal neurons.** After overexpression of HSPA12 for 24 h, the indicated gene expression was examined in hippocampus by immunoblotting analysis. n = 6/group.

Data are mean ± SD, ** $P < 0.01$ and * $P < 0.05$ by Student's two-tailed unpaired *t* test.

Figure S9. Effects of GSK-3 β on glycolytic activities in hippocampus and hippocampal neurons.

A-C. Cell experiments. Primary neurons were treated with GSK3β activator DIF3 for 3 h **(A)**. After then, cells were collected for measuring GSK-3β phosphorylation levels and expression of the indicated glycolysis-related genes using immunoblotting **(B)**. Lactate contents in culture medium was also examined **(C)**. n = 5/group

D-F. Mice experiments. After treatment with GSK3β inhibitor lithium for 10 days **(D)**, hippocampus was collected for measuring GSK-3β phosphorylation levels and expression of the indicated glycolysis-related genes using immunoblotting **(E)**. The lactate contents were examined in culture medium **(F)**. n = 3/group for immunoblotting and n = 5/group for lactate analysis.

Data are mean ± SD, * $P < 0.05$ by one-way ANOVA followed by post-hoc test. ns, no significant difference.

Figure S10. Effects of lithium treatment on dendrite length and spinogenesis of hippocampal neurons of HSPA12A knockout mice.

A. **Experimental protocol.**

B. **Neuronal dendrite length.** Neuronal dendrite length in dentate gurus was analyzed using Golgi staining. n = 6/group. Scale bar = 50 μm.

C. **Neuronal dendrite spine density.** Neuronal spine density of dendrite in dentate gurus was analyzed using Golgi staining. n = 6/group. Scale bar = 20 μm.

Data are mean ± SD, ** $P < 0.01$ by one-way ANOVA followed by post-hoc test. ns, no significant difference.

Figure S11. Re-expression of HSPA12A in hippocampus improved glycolysis and adult hippocampal neurogenesis in *Hspa12a*$^{-/-}$ mice.

A. **Gene expression.** After re-expression of HSPA12A in hippocampus of *Hspa12a*$^{-/-}$ mice, the indicated gene expression in hippocampus were examined using immunoblotting. n = 3/group.

B. **CSF lactate contents.** The lactate contents were examined in mice CSF. n = 5/group.

C. Adult hippocampal neurogenesis (AHN). AHN was examined in dentate gyrus (DG) by BrdU incorporation. DAPI was used to counter stain nuclei. The images showed the representative staining in dentate gyrus, and the boxed areas were magnified in the down panels. n = 5/group. Scale bar = 100 μm.

Data are mean ± SD; ** $P < 0.01$ and * $P < 0.05$ by one-way ANOVA followed by post-hoc test. ns, no significance.

Figure S12. Re-expression of HSPA12A in hippocampus improved behavioral alterations in *Hspa12a*$^{-/-}$ mice.

A-C. Behavioral tests. n = 8 for WT group, n = 5 for Ad-NC/*Hspa12a*$^{-/-}$ group, and n = 5 for Ad-*Hspa12a*/*Hspa12a*$^{-/-}$mice group.

Data are mean ± SD; ** $P < 0.01$ and * $P < 0.05$ by Kruskal-Wallis test. ns, no significance.
