# Supplementary material for: HSPA12A controls cerebral lactate homeostasis to maintain hippocampal neurogenesis and mood stabilization
Source: Transl Psychiatry. 2023 Aug 14;13:280. doi: 10.1038/s41398-023-02573-5 (PMC10425330; doi:10.1038/s41398-023-02573-5)
Supplement: Supplementary file 4 — Supplemental tables [file 41398_2023_2573_MOESM4_ESM.pdf]

Supplemental tables

Table S1. Antibodies used in the experiments

Antibody	Source	Company	Catalog No.
anti-HSPA12A	Rabbit	Abcam	ab200838
anti-GLUT4	Rabbit	Abcam	ab33780
anti-LDHA	Rabbit	Abcam	ab101562
anti-PKM2	Rabbit	Abcam	ab38237
anti-GADPH	Rabbit	Bioworld	AP0063
anti-GLUT1	Rabbit	Proteintech	21829-1-AP
anti-HK II	Rabbit	Cell Signaling Technology	#2867
anti- PFKFB3	Rabbit	Cell Signaling Technology	#13123
anti- GSK3β	Rabbit	Cell Signaling Technology	9315S
anti- phosphor-GSK-3β (Ser9)	Rabbit	Cell Signaling Technology	9336S
Anti-β-catenin	Rabbit	Cell Signaling Technology	9562S
anti- phosphor-β-catenin	Rabbit	Cell Signaling Technology	9561S*2
anti- BDNF	Mouse	Proteintech	66292-1-lg
anti- BrdU	Mouse	Proteintech	66241-1-Ig
anti- MCT4	Rabbit	Proteintech	22787-1-Ap
anti-β-tubulin III	Rabbit	Bioworld	AP0064
anti- NeuN	Rabbit	Millipore	ABN78
